# Tunable and Transferable Diamond Membranes for Integrated Quantum Technologies

**DOI:** 10.1021/acs.nanolett.1c03703

**Published:** 2021-12-13

**Authors:** Xinghan Guo, Nazar Delegan, Jonathan C. Karsch, Zixi Li, Tianle Liu, Robert Shreiner, Amy Butcher, David D. Awschalom, F. Joseph Heremans, Alexander A. High

**Affiliations:** † Pritzker School of Molecular Engineering, University of Chicago, Chicago, Illinois 60615, United States; ‡ Center for Molecular Engineering and Materials Science Division, Argonne National Laboratory, Lemont, Illinois 60439, United States; ¶ Department of Physics, University of Chicago, Chicago, Illinois 60615, United States

**Keywords:** diamond, color center, quantum information science, heterostructures, quantum sensing

## Abstract

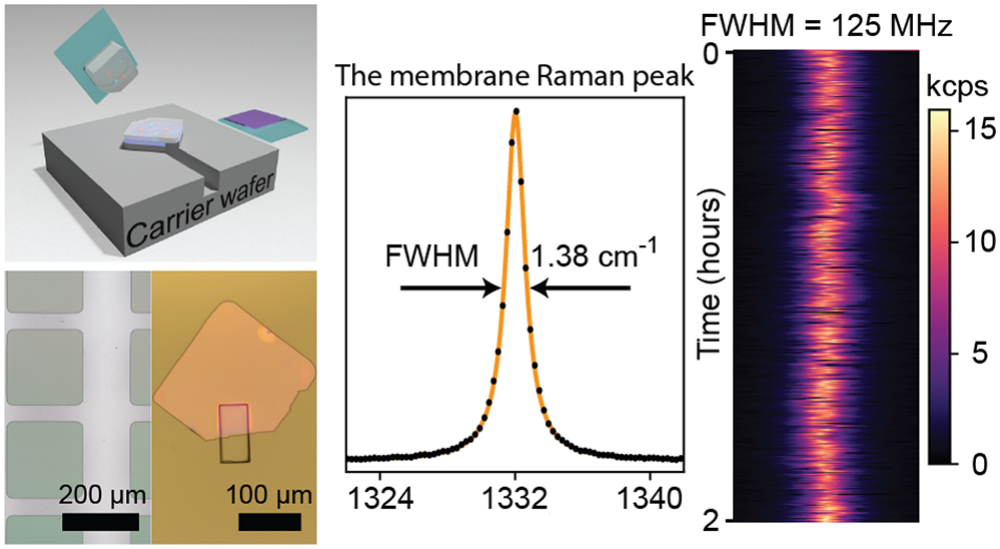

Color centers in diamond are widely explored as qubits in quantum technologies. However, challenges remain in the effective and efficient integration of these diamond-hosted qubits in device heterostructures. Here, nanoscale-thick uniform diamond membranes are synthesized via “smart-cut” and isotopically (^12^C) purified overgrowth. These membranes have tunable thicknesses (demonstrated 50 to 250 nm), are deterministically transferable, have bilaterally atomically flat surfaces (*R_q_* ≤ 0.3 nm), and bulk-diamond-like crystallinity. Color centers are synthesized via both implantation and in situ overgrowth incorporation. Within 110-nm-thick membranes, individual germanium-vacancy (GeV^–^) centers exhibit stable photoluminescence at 5.4 K and average optical transition line widths as low as 125 MHz. The room temperature spin coherence of individual nitrogen-vacancy (NV^–^) centers shows Ramsey spin dephasing times (*T*_2_^*^) and Hahn echo times (*T*_2_) as long as 150 and 400 μs, respectively. This platform enables the straightforward integration of diamond membranes that host coherent color centers into quantum technologies.

Color centers in diamond are a leading platform in quantum networking and sensing due to their exceptional coherence times,^1,2^ robust spin–photon interfaces,^3,4^ and controllable interactions with local nuclear- and reporter-spin registers.^5−7^ Color centers have been utilized in many landmark experimental demonstrations, including deterministic entanglement,^8^ multinode quantum networking,^9^ nanoscale NMR spectroscopy,^10^ and memory-enhanced quantum key distribution.^11^ These advances can be accelerated into scalable technologies with the ability to create high-quality, nanoscale-thick diamond membranes as elements in hybrid devices. Ideally, in the next generation of quantum technology, diamond will simply be another functional layer in heterostructures that can include nonlinear/magnetic/acoustic materials, on-chip detectors, superconductors, nanophotonics, and microfluidics. Such improved integration can increase entanglement efficiency,^9^ master control of phonons and photons,^11−13^ enable on-chip frequency conversion,^14^ improve interfaces with other quantum systems and sensing targets,^15,16^ and create new opportunities to engineer quantum states. However, the material properties of diamond create fundamental difficulties for heterostructure integration. Specifically, high-quality, single crystal heteroepitaxial growth of diamond thin films remains challenging despite recent progress.^17,18^ In response, a variety of processing and integration schemes have been developed to derive low dimensional structures out of bulk diamond.^19−24^ While promising, a scalable, high-yield approach that enables full heterostructure-like integration of diamond while maintaining bulk-like properties—specifically, crystallinity, surface roughness, and color center coherence—is still lacking.

Here, we report the efficient synthesis and manipulation of ultrathin diamond membranes suitable for next-generation applications in quantum information science (QIS). Our membrane fabrication procedure is based on a “smart-cut” technique,^23,25−28^ in which He^+^ implantation creates a subsurface graphitized layer that can be electrochemically (EC) etched, in conjuncture with a plasma enhanced chemical vapor deposition (PE-CVD) overgrowth optimized for the synthesis of high-quality, in situ doped, and isotopically purified diamond. The process allows detachment of arbitrarily large diamond membranes with smooth interfaces without relying on reactive-ion-etching (RIE) undercut processes. While previous works have demonstrated the effectiveness of “smart-cut” and overgrowth for diamond membrane creation, our process realizes three critical advancements to the state-of-the-art. First, we demonstrate isotopically controlled and nitrogen δ-doped diamond membrane structures with atomically flat surfaces and high crystal quality showing unprecedentedly narrow Raman line widths.^28,29^ Second, we demonstrate a novel dry-transfer technique which enables clean and deterministic membrane placement on arbitrary substrates. Membranes transferred in this way are free from premature detachment and unwieldy curvatures caused by built-in strain between the graphitized and overgrown layers.^30^ As such, these membranes can be integrated with other material platforms and functional structures. Third, we report that these structures, even at thickness ≤150 nm, are suitable hosts for germanium vacancy (GeV^–^) and nitrogen vacancy (NV^–^) centers created via implantation (in both cases) and overgrowth (NV^–^ only). Specifically, we show that the GeV^–^ centers exhibit stable and coherent emission at 5.4 K, while the NV^–^ centers show bulk-like spin coherence properties at room temperature. Additionally, we demonstrate that doping of other group IV color centers such as silicon-vacancy (SiV^–^) and tin-vacancy (SnV^–^) is also viable.

## Results and Discussion

### Diamond Membrane Synthesis

**Figure 1:**
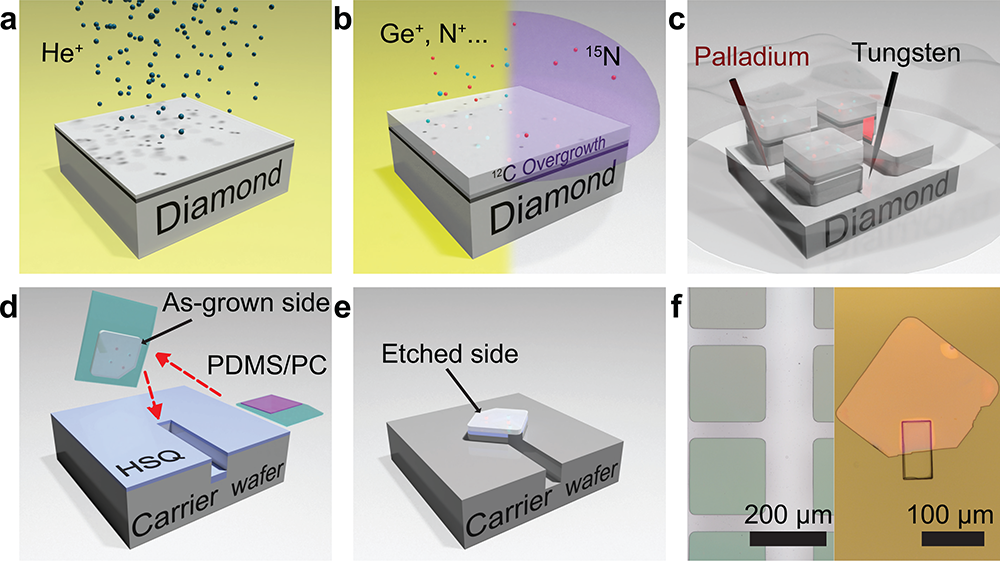
Schematics of the diamond membrane fabrication process. (a) He^+^ implantation with subsequent annealing to form the membrane (light gray on the top) and the graphitized layer (dark gray underneath). (b) Color center incorporation via either ion implantation post-isotopically (^12^C) purified overgrowth (left) or in situ doping (right). Red dots: N^+^. Blue dots: Ge^+^. Other implanted species (Si^+^, Sn^+^) are not shown. (c) Diamond membrane undercut via EC etching in DI water, with palladium anode (dark red) and tungsten cathode (dark gray) aiming at the target membrane. A transfer tether is colored red for better visualization. (d) Membrane dry transfer. The membrane is picked up by the PDMS/PC stamp (green/purple), flipped onto another PDMS stamp (green), and bonded to the carrier wafer by HSQ resist (blue). (e) Membrane back etch. The pedestal-like structure underneath the membrane is formed by ICP etching on the HSQ layer and the carrier wafer. (f) Microscope images of patterned overgrown membranes (left) and a transferred and back-etched membrane on a fused silica wafer (right). The green squares on the left are patterned membranes with underneath graphitized layer, and the rectangle on the right indicates the trench etched prior to the transfer.

The step-by-step fabrication procedure of the diamond membrane quantum platform is demonstrated in Figure 1a–e. The process starts with a low-energy (150 keV) He^+^ implantation (5 × 10^16^ cm^–2^) into diamond substrates, as shown in Figure 1a. This step forms a depth-localized graphitized underlayer ∼410 nm deep^31^ via damage-induced phase transition of the carbon bonds from sp^3^ to sp^2^. Unlike RIE-based undercut approaches,^21,22^ the top diamond layer maintains a uniform thickness and flatness throughout processing (Figure S1b and Figure S3a in SI). The substrates are then subjected to a multistep anneal (section 1.1 in SI). The high temperature allows for the mobilization and subsequent annihilation of implantation-induced crystal damage in the top layer^32,33^ as characterized via Raman spectroscopy (section 2.2 in SI). However, this process is imperfect, resulting in the top layer remaining unsuitable as a host for highly coherent color centers.

To achieve pristine crystal quality in the membranes, we follow the “smart-cut” with homoepitaxial PE-CVD of an isotopically engineered diamond thin film overlayer as shown in Figure 1b. During growth, the hydrogen to methane flow rate ratio is kept at 0.05% to ensure a morphology preserving step-flow growth regime.^1,34^ The growth rates herein were 6.2(4) nm h^–1^ to 9.3(8) nm h^–1^ for 700 °C to 500 °C heating plate temperature, respectively (section 1.2 in SI). The process can be performed with higher growth rates to efficiently achieve thicker structures. In this work, we maintained low rates compared to other works^23,26^ to ensure a more accurate depth-localization of dopant layers, i.e., δ-doping precision.^1^

We employed two distinct strategies for point defect creation within the overgrown layer. A subset of the membranes had a ∼2 nm δ-doped layer of ^15^N grown in, as schematized on the right of Figure 1b. These membranes underwent electron irradiation and subsequent annealing to form a δ-doped NV^–^ layer (section 1.2 in SI). Other overgrown diamonds were ion-implanted with Ge^+^, Si^+^, Sn^+^, and N^+^ as seen on the left of Figure 1b. These membranes were subjected to another identical multistep anneal to form optically active point defects.^32,35^

To realize a fully integrable diamond platform, we have engineered a high-yield, controllable process to lithographically pattern arbitrarily shaped membranes into the overgrown films and subsequently transfer them onto other substrates/devices. The left side of Figure 1f shows inductively coupled plasma (ICP)-defined square-shaped membrane arrays (200 μm side length) used in this work, which are of sufficient size for photonics integration.^11,13,21^ Each step of the membrane definition and transfer utilizes established cleanroom techniques (sections 1.3 and 1.4 in SI). The size and shape of the membranes are fully defined and can be tailored to specific applications, with the maximum size only limited by the substrate dimensions.

Membrane manipulation starts with an EC etching of the graphitic underlayer as shown in Figure 1c. The etching is done in deionized water (DI) by placing two electrodes close to the target membrane (≤25 μm) and applying the necessary graphite etch potential (section 1.3 in SI). Critically, in contrast to previous studies,^26,30^ a small portion of the underlayer is left unetched, creating a tether that prevents premature membrane detachment before the dry transfer. The overall efficiency to transfer membranes onto a carrier wafer is greater than 80%—mainly limited by human error during the EC etching. A single 3 mm × 3 mm diamond substrate typically results in more than 45 (200 μm × 200 μm) functional membranes.

Our transfer process, depicted in Figure 1d, draws inspiration from those utilized in van der Waals heterostructure fabrication.^36^ This process starts with a polydimethylsiloxane (PDMS)–polycarbonate (PC) stack mounted on a micropositioner with angle and position precision of 0.001° and 5 μm, respectively. This PDMS/PC stamp is used to uniformly dry-adhere and subsequently break-off the target membrane from the tether. The membrane is then flipped with another PDMS stamp and placed on a hydrogen silsesquioxane (HSQ)-coated carrier wafer (Figure S4 in SI). Next, the structure is annealed at 600 °C to allow the HSQ to transition into a denser, low thermal expansion coefficient,^37^ low optical loss,^13^ photochemically inert, and easy-to-process SiO_*x*_ film.^38^ The success of this process is underpinned by the adhesion differences between PC, PDMS, and HSQ layers. This guarantees a transfer that is highly deterministic, reproducible, and agnostic to carrier wafer and surface patterning. The overall height variation across the whole membrane is determined to be σ ≤ 10 nm (Figure S5e in SI). In this work, we bond diamond membranes to fused silica and thermal oxide wafers with predefined trenches, generating locally suspended regions. Suspension allows us to control the chemical termination of both surfaces and reduce HSQ-related fluorescence for photoluminescence (PL) characterizations of the embedded point defects (sections 1.8 and 2.4 in SI).

Finally, by flipping the membrane, we are able to fully etch away the He^+^-damaged, graphitized diamond layer with chlorine-based ICP, as shown in Figure 1e. This ICP etching eliminates the undesired fluorescence and built-in strain caused by crystallographic imperfections and lattice mismatch,^27^ while tuning the final membrane thickness (section 1.4 in SI). The right part of Figure 1f shows a 20 h grown diamond membrane on a fused silica wafer with 100 nm final thickness. As such, this membrane synthesis approach provides a clear, high yield, scalable, and easy path forward for generating multifield relevant diamond membranes.

### Surface and Material Quality

**Figure 2:**
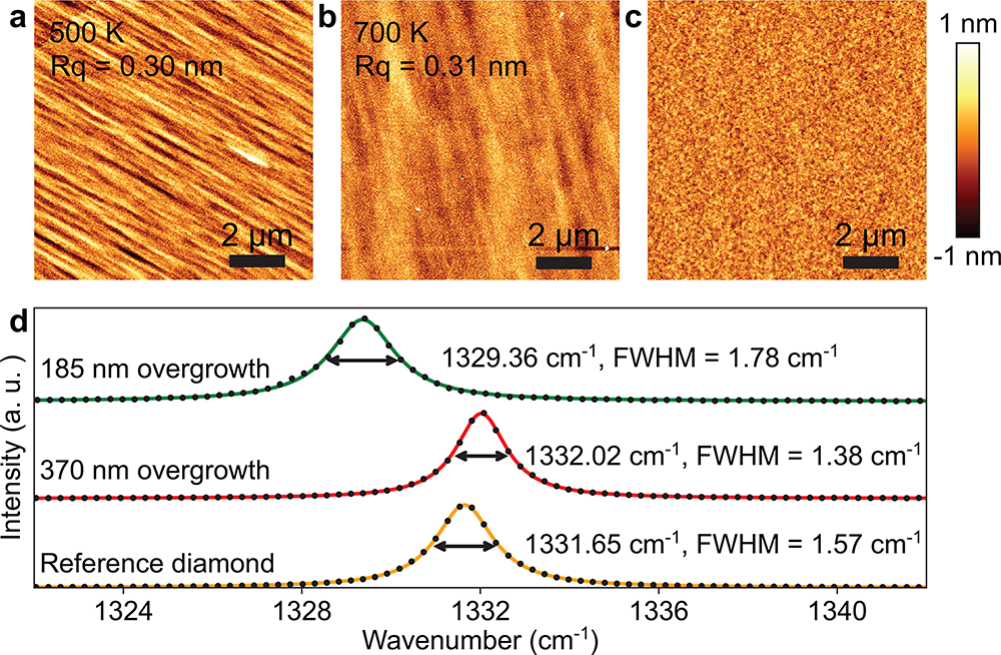
Surface morphology and crystal quality of overgrown membranes. (a,b) AFM images of overgrowth patterns (as-grown side of membranes) at different heating plate temperatures. (c) AFM image on the etched side of the membrane after multistep etching. (d) Room-temperature Raman spectroscopy of diamond membranes and the reference diamond substrate. Green: ∼185 nm (20 h) overgrowth membrane back-etched down to 100 nm. Red: ∼370 nm (40 h) overgrowth, isotopically purified membrane back-etched down to 110 nm. Yellow: Surface strain-released, EL-grade single crystal diamond used as the reference. The surface strain is polish-induced, and is removed by ICP etching and subsequent annealing prior to the Raman spectroscopy (section 2.2 in SI).

The utility of diamond membranes in QIS demands exceptional surface and material quality. Figure 2 shows the atomic force microscopy (AFM) characterizations of the membrane as-grown side (a,b) and etched side (c) topology following all processing. We note that for both growth conditions (500 °C for 20 h and 700 °C for 40 h), the resulting surface is smooth, showing distinct step-flow growth striations,^39^ with a roughness (*R*_q_ ≤ 0.31 nm) lower than the diamond lattice constant (0.357 nm). Similarly, the etched side, although initially rough due to the intrinsic straggle of the He^+^ implantation process (Figure S1e in SI), reaches *R*_q_ of ≤0.3 nm following ICP etching,^40^ as shown in Figure 2c. The realization of atomically flat surfaces is critical for effective surface termination and coherence protection of near-surface color centers.^32^

Additionally, the diamond crystal quality was investigated via room-temperature Raman spectroscopy as shown in Figure 2d. A surface strain-released^35^ reference diamond (single-crystal, electronic (EL) grade) from Element Six, with a Raman line width of 1.570(1) cm^–1^, was used as a comparison benchmark. The ∼185 nm overgrowth membrane (20 h growth in 500 °C, back-etched down to 100 nm) presents a Raman line width of 1.779(5) cm^–1^, slightly larger than the reference value. Remarkably, the isotopically purified, ∼370 nm overgrowth membrane (40 h growth in 500 °C, back-etched down to 110 nm) presents a line width of 1.375(2) cm^–1^ (1.486(14) cm^–1^ for the ∼250 nm isotopically purified overgrowth at 700 °C, Figure S2b in SI), significantly lower than anything (including bulk diamond) reported previously.^28^ The ultranarrow Raman peak indicates that the crystal is free of impurities and defects. The up-shifting and narrowing, in comparison with the bulk spectra, are consistent with the change of the Raman transition brought on via isotopic purification of a high-quality diamond structure.^29^ As such, the Raman spectra indicate that these diamond membranes are of ideal crystal quality to host color centers.

### Implanted Group IV Color Centers

**Figure 3:**
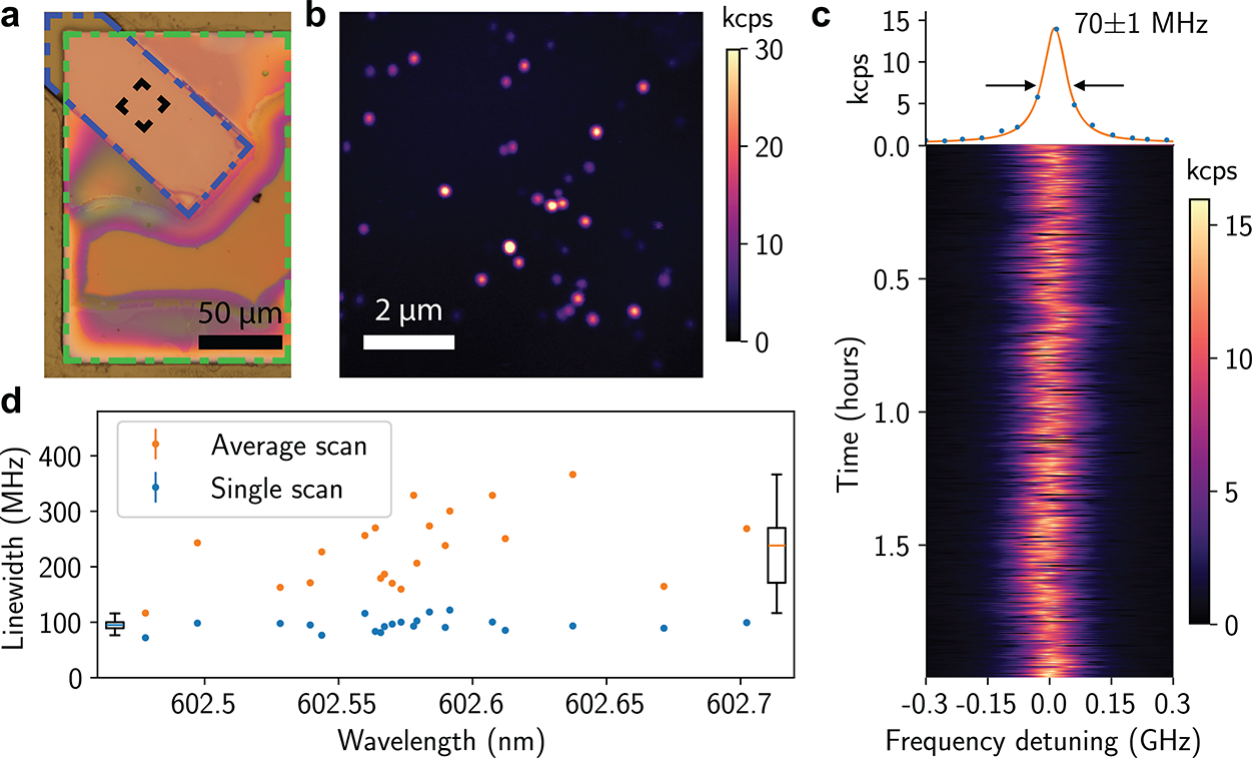
Optical characterization of GeV^–^ centers at 5.4 K. (a) Microscope image of a diamond membrane (dashed green) containing implanted GeV^–^ centers. HSQ in the trench (dashed blue) is completely removed by vapor HF. The rainbow color on the membrane indicates excess HF undercut (section 2.4 in SI). The PL measurement is performed in the dashed black region. (b) PL map of the implanted GeV^–^ centers. The signal-to-background ratios for most centers are between 5 to 30. (c) Single PLE scan and time-averaged broadening of a GeV center. The data sampling time per point is 100 ms, and the counts are normalized to kilo-counts-per-second (kcps). (d) Statistics of GeV^–^ optical line widths measured via single-scan (blue) and average broadening (orange). The inset box plot on the left (right) indicates the median single (average) line width of 95 MHz (231 MHz).

In order to investigate the coherence of color centers in the membranes, we performed detailed measurements of the optical (spin) coherence of GeV^–^ (NV^–^). We mounted the 110-nm-thick (∼370 nm overgrowth) ^12^C-membrane on a trenched thermal oxide wafer with a vapor HF-based HSQ removal (section 2.4 in SI), as shown in Figure 3a. This HSQ removal is optional, and the additional HSQ fluorescence has minimal impact on device performance for GeV^–^ resonant experiments. GeV^–^ centers inside the sample were subsequently characterized at 5.4 K. This particular membrane was also implanted with nitrogen (further in the text), silicon, and tin (section 1.5 in SI).

A PL map of the GeV^–^ centers over the suspended area is shown in Figure 3b. The GeV^–^ conversion efficiency was determined to be 6.5(4) % (section 3.2 in SI). Typical zero phonon line (ZPL) peaks of GeV^–^ centers lie between values reported in bulk diamond,^41,42^ indicating a homogeneous crystal environment (Figure S6a in SI). The span of the ZPL peaks across different centers come from the nature of the implantation.^43^ The inhomogeneous broadening of the ZPL positions are comparable with those measured in bulk diamond under identical implantation and annealing conditions. While strain was generated from the membrane mounting, thermal expansion ratio mismatch with the carrier wafer, and HSQ annealing may potentially contribute to energetic variation, our measurements indicate that the crystal environments between the diamond membrane and the bulk diamond are highly similar (section 3.5 in SI). Both off- and on-resonance autocorrelation measurements were performed on the GeV^–^ centers (section 3.3 in SI), confirming antibunching features associated with single photon emitters with *g*^(2)^(0) = 0.17(2) and 0.19(5), respectively. Additionally, the transition line widths of the GeV^–^ centers are relatively low, with single scan lines as narrow as 70(1) MHz. Furthermore, this optical transition is stable, offering a 2 h average spectral broadening as low as 124.8(2) MHz, as seen in Figure 3c. This broadening is still less than a 5-fold increase over the intrinsic line width.^41^

For completeness, we also analyzed the statistical distribution of single-scan line widths and time-averaged broadening. Out of 38 centers observed with off-resonant excitation, we were able to resolve the C transition in 29 centers with resonant scanning. Additionally, in 7 (1) centers, we observed the C transition switches between 2 (4) stable peaks (section 3.4 in SI). The origin of this switching between two stable energies, distinct from random spectral diffusion, is the subject of ongoing study. Statistics of the other 21 single-peak GeV^–^ centers yield a median single (2.5 min average) scan line width of 95 MHz (231 MHz), as shown in Figure 3d. Recent cavity quantum-electrodynamics measurements^44^ have realized a spin-photon coupling rate of *g* = 2π × 5.6 GHz, significantly larger than the observed spectral broadening in our centers. Therefore, the membrane-hosted GeV^–^ centers are sufficiently optically coherent for advanced applications in quantum networking and entanglement generation. These diamond membranes are also viable hosts for SiV^–^ and SnV^–^ centers (section 3.3 in SI).

### Embedded NV^–^ Centers

Next, we investigated the spin coherence of NV^–^ centers in the membranes using a home-built room temperature PL microscope (section 4.1 in SI). In experiments that utilize group IV diamond color centers as spins, the Zeeman frequencies are typically within an order of magnitude of the NV center spin splitting,^2,45,46^ and NV^–^ coherence measurements reveal magnetic noise levels which are also relevant to group IV coherent control.^32,47−49^ All NV^–^ center measurements presented herein were performed with a 15 gauss static magnetic field applied at 10° angle to the [111] crystal axis. We measured NV^–^ centers along all four possible crystal orientations, determined by different transition frequencies.

**Figure 4:**
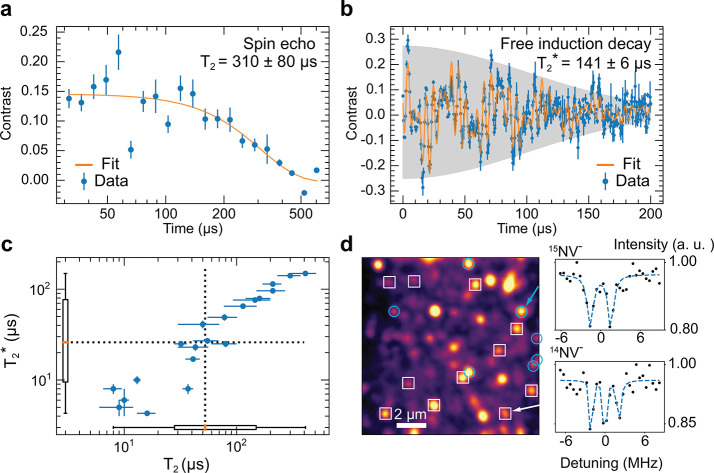
Optical characterization of embedded NV^–^ centers at room temperature. (a,b) Representative spin echo and free induction decay curves on a single long-lived NV^–^ spin, accompanied by the *T*_2_^*^ and *T*_2_ coherence times. A detailed description of data analysis is provided in the SI. The oscillations in the first 100 μs of (b) arise from aliasing of electron spin echo envelope modulation. (c) Scatter plot of *T*_2_^*^ and *T*_2_ times for the 20 measured NV^–^ centers. Inset box plots denote median values of 26 and 52.5 μs (dashed lines) and lower-quartile values of 9.5 and 28 μs. Error bars are fit errors. (d) NV^–^ PL map of a δ-doped membrane with ^15^NV^–^ centers (teal circles) and ^14^NV^–^ centers (white squares) labeled. At right, pulsed-ODMR spectra of the indicated NV^–^ centers.

Figure 4a,b shows representative free induction decay and spin echo decay curves on a single long-lived NV^–^ spin, with the fitted *T*_2_^*^ and *T*_2_ coherence times. The oscillations in the first 100 μs of Figure 4b arise from aliasing of electron spin echo envelope modulation (a finer trace to demonstrate the origin of this is presented in the Figure S7c of SI). Together, these measurements demonstrate that membrane fabrication does not preclude the formation of highly coherent spin qubits. Figure 4c presents a scatter plot of the *T*_2_^*^ and *T*_2_ times, showing a spread of 4.3(3) μs to 149(7) μs and 8(2) μs to 400(100) μs, with most times (median 50%) falling above 10 and 30 μs, respectively.

Comparing the background nitrogen concentration (section 4.5 in SI) and the observed areal NV^–^ density versus the [*N*] implantation dose of 2 × 10^8^ cm^–2^, we expect that many of our observed NV^–^ formed from in-grown nitrogen and vacancies introduced during ion implantation and not exclusively from the implanted nitrogen. Thus, the observed NV^–^ are likely distributed throughout the thickness of the membrane, with some residing within ≤15 nm of both surfaces, where previous work demonstrated marked decoherence from surface noise.^48^ However, statistically, the surface proximity distribution of the NV^–^ alone cannot fully account for the large number of NV^–^ centers with *T*_2_ ≤ 100 μs. The multispecies implantation process is known to introduce crystal damage throughout the ion path, which can create spin-full vacancy complexes that are neither mobilized nor annihilated during the annealing process.^49^ It is likely that the resulting inhomogeneity of the bulk spin bath is the main factor limiting NV^–^ coherence times. Nonetheless, the spin echo coherence time (*T*_2_ up to 400 μs) is competitive with near bulk-like properties, and the free induction decay (*T*_2_^*^ up to 150 μs) outperforms commercially available bulk material due to the ^12^C purification. Therefore, the coherence times presented herein are fully compatible with applications in quantum sensing and hybrid quantum systems.^47,50,51^

This membrane fabrication technique is also highly amenable to in situ δ-doping of ^15^N during overgrowth.^1^ δ-Doping allows deterministic incorporation of dopants (N, Ge, Si, etc.) during membrane overgrowth while providing a valuable distinction from the intrinsic, isotopically naturally abundant defects (i.e., isotopically incorporating ^15^N during growth to distinguish from the ^14^N overgrowth background). As a proof of concept, we introduced 2-nm-thick area of ^15^N doping ∼36 nm from the as-grown side of a 110-nm-thick diamond membrane (∼250 nm overgrowth at 700 °C). Figure 4d shows a PL map of NV^–^ centers in such a sample. The ^15^NV^–^ centers are labeled in teal circles, while background ^14^NV^–^ are in white rectangles, with representative hyperfine-resolved ODMR spectra presented to the right of the figure. We observed a 7:11 ratio of [^15^NV^–^]:[^14^NV^–^] (SIMS characterized [^15^NV^–^] of 30.8(57) ppb, section 4.5 in SI). This is in a good alignment with what was observed for the implantation-synthesized NV^–^ centers, showing a consistent background ^14^NV^–^ density throughout the membrane from the overgrowth process. A rigorous quantitative comparison of optimized implanted and in-grown defects as they relate to the membrane surfaces proximity is left for subsequent studies.

## Conclusion

We have created uniform, tunable, transferable, large-scale diamond membranes with material and surface quality comparable to and even exceeding that of bulk diamond. Additionally, we have demonstrated that color centers within the membranes have sufficient optical and spin coherence for a broad range of applications in QIS. These diamond membranes can serve as a quantum material platform for a wide range of current and future research directions. The subwavelength thickness of the diamond membranes expands capabilities for nanophotonic integration.^13^ Additionally, this platform eases the fabrication of strain engineering^52^ and Stark tuning,^53^ which also provides an opportunity to study the behavior of color centers under extreme environments such as strong strain or electrical fields. Moreover, the versatility afforded by the arbitrary carrier wafer platform is vital for integrating diamond-based quantum systems to phononic,^12,54^ superconducting, and magnonic^55^ systems for quantum transduction in a hybrid geometry.^56^ Furthermore, NV^–^-based quantum sensing will benefit from the ease of integration with a number of interfaces, ranging from type-II superconductors to living cells.^15,16^ The isotopically purified, highly crystalline diamond membranes are also prime candidates for heat dissipation device studies^57^ and high-quality engineered nanodiamond synthesis.^58,59^

## Supporting Information Available

Details of diamond membrane synthesis, material characterizations (AFM, Raman, SIMS, etc.), optical setups, and additional analysis of GeV^–^ and NV^–^ measurements (PDF)
