# Supplementary material for: Tunable and Transferable Diamond Membranes for Integrated Quantum Technologies
Source: Nano Lett. 2021 Dec 13;21(24):10392–9. doi: 10.1021/acs.nanolett.1c03703 (PMC8704172; doi:10.1021/acs.nanolett.1c03703)
Supplement: Supplementary file 1 — nl1c03703_si_001.pdf [file nl1c03703_si_001.pdf]

Supporting Information: Tunable and Transferable Diamond Membranes for Integrated Quantum Technologies

Xinghan Guo[1], Nazar Delegan[1,2], Jonathan C. Karsch[1], Zixi Li[1],
Tianle Liu[3], Robert Shreiner[3], Amy Butcher[1], David D. Awschalom[1,2,3],
F. Joseph Heremans[1,2], Alexander A. High[1,2*]
[1]*Pritzker School of Molecular Engineering, University of Chicago, Chicago, IL 60637, USA*
[2]*Center for Molecular Engineering and Materials Science Division,*
Argonne National Laboratory, Lemont, IL 60439, USA
[3]*Department of Physics, University of Chicago, Chicago, IL 60637, USA*
[*]*E-mail: ahigh@uchicago.edu*

1 Diamond membrane fabrication procedure

1.1 Membrane synthesis via the graphitized underlayer formation

Standard $3\,\mathrm{mm}$ by $3\,\mathrm{mm}$ by $0.25\,\mathrm{mm}$ single crystal, optical grade diamond substrates (Element Six, $\leq 1\,\mathrm{ppm}$ [N]) are used for the membrane synthesis. These are initially fine-polished to a surface Rq of $\leq 0.3\,\mathrm{nm}$ (Syntek LLC.) as to minimize morphological inconsistencies (see Figure S1 (a)). Next, the samples are implanted with $^{4}He^{+}$ ions (CuttingEdge Ions LLC.) at $150\,\mathrm{keV}$ to create a graphitized layer at $\approx 410\,\mathrm{nm}$ depth. This is done at an incidence angle of $7°$ to avoid ion channeling. The dose is set to $5 \times 10^{16}\,\mathrm{cm}^{-2}$, which is closely above the graphitization threshold as to minimize crystal damage (see section 1.5). In this work, a three-step annealing process was employed after the implantation: a $400\,°\mathrm{C}$ soak for $8\,\mathrm{h}$, followed by a $800\,°\mathrm{C}$ soak for $8\,\mathrm{h}$, completed with a $1200\,°\mathrm{C}$ anneal for $2\,\mathrm{h}$.[1] This was done in a forming gas environment ($Ar{:}H_2$ of $96 : 4$). The implantation and annealing had no negative impact on the surface roughness (see Figure S1 (b)). The phase transition of the carbon bonds during membrane formation is studied via room temperature Raman spectroscopy (see section 2.2).

1.2 PE-CVD diamond overgrowth and δ-doping of ^{15}N

Homoepitaxial PE-CVD of diamond is performed in a custom configured *SEKI DIAMOND SDS6350* system. Before loading into the growth chamber, the diamond substrates are chemically tri-acid cleaned (see section 1.7). The diamond sample is placed on a grooved molybde-

num substrate to keep it flat with the rest of the plasma exposed surface, allowing for a uniform growth rate across the sample, while minimizing edge effects. The chamber is pumped down to a 2×10^{-8} Torr to 5×10^{-8} Torr base pressure in order to reduce background contamination. Thereafter, high purity H_2 (99.999 999 % chemical purity) is introduced into the chamber, with the gas flow ratios constant and pumping rate monitored as to maintain a constant process pressure of 25 Torr. Throughout the process the microwave power is maintained at 900 W ($11.5\,\mathrm{W\,mm^{-2}}$). Before introducing the carbon precursor, the substrate is heated to a 500 °C or 700 °C setpoint which translates to a pyrometer assessed plasma temperature of 540(10) °C or 740(10) °C, respectively. The H_2-only plasma conditions are maintained for 20 min (500 °C) and 1 h (700 °C) as to etch away any residual surface carbonaceous contaminants – the approximated sp^3 etch depth is ≤1 nm.[2] Thereafter, $^{12}CH_4$ (99.999 99 % chemical purity, 99.99 at.% isotopic purity) is introduced as the carbon precursor, this injection is substantiated in-situ via increase of the pyrometer detected substrate surface temperature to 765(10) °C (580(10) °C for the 500 °C set-point grown substrate). The methane-to-hydrogen ratio is maintained constant at 0.05 % (H_2:$^{12}CH_4$ = 400 sccm : 0.2 sccm) as to ensure step-flow growth.[3,4] Growth rates for the 700 °C and 500 °C membranes were determined to be $6.2(4)\,\mathrm{nm\,h^{-1}}$ and $9.3(8)\,\mathrm{nm\,h^{-1}}$, respectively (see section 2.3). The reduction in growth rate is explained by an increased surface mobility and desorption of precursor adatoms[5] and likely due to a non-linear reduction of nucleation sites.

In-situ nitrogen doping is accomplished by introducing 0.06 sccm of $^{15}N_2$ gas (99.99 % chemical purity, 99.9 at.% isotopic purity) for 2 min during the growth phase. This creates a 36 nm depth localized layer with a SIMS quantified [$^{15}NV^-$] density of 30.8(57) ppb (see section 4.5). Thereafter, a cap layer is overgrown to encapsulate the δ-doped layer as deep in the membrane as desired. Subsequent irradiation and annealing allows the conversion of this layer into ≈0.9 ppb of [$^{15}NV^-$], resulting in an average color center creation efficiency of ≤2.5 %, in agreement with previous studies (see section 4.5).[3]

1.3 Membrane patterning and EC etching

Atomic layer deposition (ALD) is used to deposit 25 nm of Al_2O_3 on the overgrown membrane samples. This oxide layer serves as selective hard mask for subsequent ICP etching. Thereafter, a photoresist layer (AZ MiR 703) is applied to the sample, followed by a lithography exposure (Heidelberg MLA150, 375 nm laser, with a $150\,\mathrm{mJ\,cm^{-2}}$ dose). A total of 56 square-shaped membranes are patterned on a single substrate. Next, the sample is developed (AZ 300 MIF) and two-step etched in a chlorine-based ICP chamber (Plasma - Therm, Al_2O_3 etched with BCl_3 chemistry first, with diamond subsequently etched in O_2 chemistry, see Table S2). This step exposes the underlying graphitic layer. After etching, the sample is ultrasonicated in 80 °C N-Methyl-2-pyrrolidone (NMP) solution and placed in a buffered oxide etch (BOE) solution to remove excess resist and oxide layers.

For the EC etching step, the sample is held by double side tapes at the bottom of a DI water-filled petri dish which is mounted on a probe station (Signatone S1160). Unlike previous studies which required customized platinum tips,[6] we use commercially available tungsten tips (Signatone SE-T) as the cathode and palladium tips (Signetone SE-P) as the anode (see Figure S3

(b)). These tips are mounted to 3-axis micropositioners (Signatone S926), which are connected to a DC power supply (B&K Precision 1787B). Typically, a voltage between $15\,\mathrm{V}$ and $36\,\mathrm{V}$ is applied to undercut the membranes, with larger undercut areas requiring larger potentials and longer time. The EC etch is visually tracked, with the voltage cut at the moment that the desired undercut tether has been achieved. This process, while relatively simple, requires constant supervision as the final tether feature size is typically less than $50\,\mathrm{\mu m}$ in any direction. Practically speaking, at the typical voltages quoted above, this translates to an etching time of $\approx 30\,\mathrm{min}$ per membrane (see Figure S3 (c)). This processing step is a great candidate for future automation.

1.4 Membrane transfer and post-processing

Prior to the membrane dry transfer, the intended carrier wafers or devices are prepared with pre-defined structures and features (see section 1.8). In this work, we use $7\,\mathrm{mm}$ by $7\,\mathrm{mm}$ fused silica (SiO_2, JGS2 grade) and thermal oxide ($280\,\mathrm{nm}$ $SiO_2{-}Si$) wafers. Alignment markers and $5\,\mathrm{\mu m}$ deep trenches are patterned and etched into substrates to suspend part of the transferred membrane (see Figure S5 (a)). In this way, both surfaces of the membranes are exposed, enabling double-sided surface termination via wet-chemical or dry processes with acid cleaning and oxygen annealing used in this work. As for the dry-transfer itself, we start by mounting the diamond substrate on a transfer station rotational chuck (Signatone S1160). Parallel to this, we prepare a PDMS/PC stamp on a glass slide and mount it into a computer-aided micropositioner (Signatone CAP - 946). Next, we slowly bring the stamp down and allow it to fully adhere to the target membrane. With a quick movement of the now-adhered stamp, we break the membrane from the tether. Then, we replace the substrate on the chuck with a second PDMS stamp on a glass slide. The detached membrane can be transferred to this second stamp by slowly bringing the PDMS/PC stamp into contact with the "stickier" PDMS-only stamp and lifting the PDMS/PC stamp afterwards. Finally, with the membrane fully adhered to the PDMS-only stamp, we use the micropositioner to mount the membrane to an HSQ-covered carrier wafer on the chuck. After careful alignment, we gradually bring the stamp down until the membrane is fully attached to a freshly spin-coated HSQ layer ($14\,\%$ HSQ in methyl isobutyl ketone (MIBK) solution, DisChem Inc.), and then slowly release the stamp. The membrane is left on the carrier wafer due to stronger adhesion from the HSQ polymer and the residual MIBK solvents (see Figure S4).

After membrane transfer, the carrying wafer is annealed at $600\,°\mathrm{C}$ for $8\,\mathrm{h}$ in Ar environment. The HSQ adhesion layer fully collapses during the annealing and forms a nearly strain-free $\approx 250\,\mathrm{nm}$ thick layer. This thickness can be tuned by using different concentration of HSQ solvents or by varying the spinning parameters. In the case of partially suspended membranes, we applied a vapor HF treatment (Memsstar ORBIS ALPHA) to fully remove the HSQ in the trench (see section 2.4). Additionally, in order to etch the original damaged underlayer (the etched side of the membrane), improve surface morphology, and tune the final thickness, we submit the mounted membrane to a three-step ICP process (see Table S2).[1] We start with the "Ar/Cl_2" recipe ($2\,\mathrm{min}$ to $5\,\mathrm{min}$, depending on the target thickness) to polish the surface, then we use the "O_2/Cl_2" recipe ($30\,\mathrm{s}$ to $90\,\mathrm{s}$) to remove most of the chlorine-based compounds on the diamond surface. Finally, we apply a $30\,\mathrm{s}$ "O_2" etch to remove the residual chlorine compounds

and correctly terminate the surface. In order to avoid chemical contamination, etching steps are separated by multiple pump-purge cycles. AFM characterizations are added post-etching to ensure no micromasking occured during ICP processes.[7] The membrane thickness is monitored via either a profilometer (Bruker DektakXT) or an ellipsometer (Horiba UVISEL2) (see section 2.3).

1.5 Additional information on ion implantation

Two distinct ion implantation runs were performed in this work: $^{4}He^{+}$ ion implantation for the creation of the graphitized underlayer and $^{14}N^{+}$, $^{28}Si^{+}$, $^{74}Ge^{+}$, and $^{120}Sn^{+}$ ion implantation for the creation of color centers. Both implantation processes were performed at CuttingEdge Ions, LLC.

To achieve the formation of the graphitized underlayer, a low energy $^{4}He^{+}$-ion implantation into the diamond substrates was done as depicted in Figure 1 (a). This step forms a depth-localized graphitized underlayer $\approx$410 nm deep via a damage-induced phase transition from sp^3 to sp^2. The implantation energy was kept at 150 keV to minimize crystal damage along the $^{4}He^{+}$ trajectory. The dose was set to $5 \times 10^{16}\,\mathrm{cm}^{-2}$, which is above the graphitization threshold, but minimized to avoid unnecessary crystal damage. Separate implantation tests showed that lower doses of $\leq 2 \times 10^{16}\,\mathrm{cm}^{-2}$ were insufficient to generate a reliable graphitized layer, with the diamond substrate remaining transparent. Therefore, the desired damage threshold was established to be between $2 \times 10^{16}\,\mathrm{cm}^{-2}$ to $5 \times 10^{16}\,\mathrm{cm}^{-2}$.

We use the Stopping and Range of Ions in Matter software (SRIM)[8] to estimate the ion implantation depth and straggle. For the simulations, the density of carbon is set to $3.51\,\mathrm{g\,cm}^{-3}$, and the incidence angle is 7° to avoid ion channeling. For color center incorporation, we simultaneously implanted $^{14}N^{+}$, $^{28}Si^{+}$, $^{74}Ge^{+}$ and $^{120}Sn^{+}$ at a dose of $2 \times 10^{8}\,\mathrm{cm}^{-2}$ in order to achieve an individual-level color center density. Details about the implantation parameters and simulated depths and straggles are shown in Table S1.

All of the implantation steps were followed with the three-step 1200 °C anneal[1] (see section 1.1) to form the graphitized layer and color centers, in the case of the He^{+} and color center implantation, respectively.

1.6 Electron irradiation

For the synthesis of delta-doped color centers, an electron radiation of $10 \times 10^{18}\,\mathrm{cm}^{-2}$ at 2 MeV at the low energy accelerator facility (LEAF) at Argonne National Laboratory was performed to form vacancies throughout the diamond crystal. This was followed by a 6 h vacancy mobilization anneal at 850 °C in forming gas (96 : 4 of $Ar:H_2$).

1.7 Surface cleaning and termination

In order to remove contamination and passivate the surface, acid-related surface cleanings and terminations are applied multiple times during membrane synthesis. Firstly, diamond substrates receive a tri-acid cleaning (1 : 1 : 1 nitric, sulfuric, and perchloric acid) at 200 °C for 2 h right

before the overgrowth to eliminate surface contamination[9]. Secondly, the diamond membrane template receives a di-acid cleaning ($1:1$ nitric and sulfuric acid) at $225\,^{\circ}\mathrm{C}$ for $2\,\mathrm{h}$ after every transfer step to prevent accumulated contamination induced by EC etching or PDMS/PC stamping. Finally, before optical measurements, membrane samples receive another di-acid cleaning and an optional oxygen termination, depending on the type of color centers being characterized. For group IV centers, we only apply the di-acid clean procedure. For NV^- center measurements, an additional oxygen termination is applied following di-acid cleaning, which includes a $4\,\mathrm{h}$ annealing at $400\,^{\circ}\mathrm{C}$ in O_2 atmosphere, with $5\,\mathrm{min}$ rinse in freshly prepared Piranha solution ($3:1$ sulfuric acid and hydrogen peroxide) before and after the annealing. The oxygen termination is intended for optimizing the NV^0 to NV^- conversion and stability[10]. The incorporation of higher temperature oxygen annealing while avoiding surface etching is an ongoing study and beyond the scope of this work.

1.8 Carrier wafer preparation

In this work, we dice thermal oxide and fused silica wafers to $7\,\mathrm{mm}$ by $7\,\mathrm{mm}$ squares (Disco DAD3240) as carrier wafers. Some wafers receive $\approx 5\,\mathrm{\mu m}$ trench fabrication on the surface to allow membranes to receive double-side surface treatment and obtain low background fluorescence during optical measurements.

In order to etch the trenches, we deposit a $\approx 6\,\mathrm{\mu m}$ photoresist layer (AZ 4620) as the etching mask. The spinning speed is set to $500\,\mathrm{rpm}$ for $10\,\mathrm{s}$ and then $4000\,\mathrm{rpm}$ for $50\,\mathrm{s}$, with a ramping rate of $100\,\mathrm{rpm\,s^{-1}}$ and $2000\,\mathrm{rpm\,s^{-1}}$, respectively. After resist spinning and pre-baking, the sample is lithographically exposed (Heidelberg MLA150, $405\,\mathrm{nm}$ laser with $320\,\mathrm{mJ\,cm^{-2}}$ dosage), followed by development in AZ 400K 1:4 solution. We use fluorine-based ICP (Plasma - Therm) for SiO_2 and Si etching. After etching, samples are ultrasonicated in $80\,^{\circ}\mathrm{C}$ N-Methyl-2-pyrrolidone (NMP) solution to remove the resist.

1.9 Additional discussions on diamond membrane fabrication

The membrane creation process highlighted herein advances scalable diamond quantum research in several important ways. While the process requires initial diamond substrates with sufficient crystallinity and smoothness, it is agnostic to moderate impurity and defect concentrations within the substrate. This eliminates the need for highly purified bulk diamond substrates, which are costly and scarce. Additionally, while bulk diamond crystals are commercially grown and available for purchase in a limited range of dimensions, the size and the shape of the membranes are fully defined and can be tailored to specific applications, with the maximum size only limited by the substrate dimensions. While our choice of membrane size ($200\,\mathrm{\mu m} \times 200\,\mathrm{\mu m}$) is well motivated by scientific applications, such delaminations over millimeter size have been achieved[11], and there are no technical barriers to realizing larger dimensions with our technique.

2 Material characterization

2.1 AFM characterization

We extensively apply AFM measurements (Bruker Dimension Icon) to monitor the surface morphology throughout the synthesis steps. The scanning ranges and resolutions in both directions are set to $10\,\mu m$ and 512 lines (except for Figure S1 (f), $5\,\mu m$ with 256 lines) to observe the surface morphology and growth- or etching-related features.

The surface maps of overgrown and back-etched diamond membranes are shown in Figure 2 (a-c) of the main text, with maps of other key steps shown in Figure S1. Since the as-purchased diamond substrates are only roughly polished ($Ra \leq 30\,\mathrm{nm}$), we applied another fine-polish (Syntek LLC.) step to reduce the roughness to $Rq = 0.30\,\mathrm{nm}$ ($Ra = 0.20\,\mathrm{nm}$), as shown in Figure S1 (a). The final surface miscut, relative to the crystallographic (001) axis, is specified to $\leq 3°$. Unfortunately, while this value is relatively consistent within a single substrate batch, it can vary between substrate lots, affecting the effective implantation angle and overgrowth characteristics[4].

The He^+-graphitized and subsequently annealed substrate is shown in Figure S1 (b), with the same level of surface morphology ($Rq = 0.27\,\mathrm{nm}$). Figure S1 (c) exhibits the $40\,\mathrm{h}$ overgrown diamond surface with a pre-growth $60\,\mathrm{min}$ hydrogen plasma etch. The inconsistency of surface roughness under the same duration of hydrogen plasma etch (see Figure 2 (b)) may come from the residual surface strain from fine polishing. A surface strain release step[1] after the fine-polish should improve this in future iterations of the fabrication process. Another $40\,\mathrm{h}$ growth without proper surface preparation is shown in Figure S1 (d). Residual contamination on the diamond surface generates growth defects (such as pits or pyramids) along the process. Figure S1 (e) shows the etched side of the diamond membrane after the dry transfer. The excess roughness ($Rq = 1.17\,\mathrm{nm}$) comes from the straggle of the He^+ implantation and can be reduced by subsequent "Ar/Cl_2" etching. It is crucial to apply "Ar/Cl_2" to prevent roughness deterioration. Figure S1 (f) shows the etched side of the membrane if the O_2 etching is applied directly, where the surface deteriorates noticeably due to the preferential etching nature of an O_2-based plasma on crystallographic defects and polish-induced damage.

2.2 Raman characterization

Throughout the process, we applied Raman spectroscopy (Horiba Scientific LabRAM HR Evolution) to investigate both the phase of the carbon during membrane formation and the diamond crystal quality. A $633\,\mathrm{nm}$ excitation laser is used instead of the more popular $532\,\mathrm{nm}$ to avoid broad fluorescence from NV^- centers in the substrates. We use the finest grating ($1800\,\mathrm{gr\,mm^{-1}}$) for data collection, with a quoted resolution of $\approx 0.3\,\mathrm{cm^{-1}}$. The resulting peak position uncertainty is caused by this instrument resolution, while the linewidth uncertainty comes from the fittings. The pinhole of the Raman confocal microscope is set to either $30\,\%$ or $50\,\%$ to reduce artificial broadening.

To effectively evaluate the crystal quality of our overgrown membranes, we used a reference diamond for the Raman measurements. In order to achieve the cleanest Raman signature, we

chose an electronic grade single crystal diamond from Element Six. Furthermore, we ICP-etched the top $\approx 5\,\mu m$ of the reference sample, and subsequently annealed the substrate in order to remove the residual strain induced by surface polish. The etching procedure is described in [12], with our exact process flow detailed in Table S2.

Figure S2 (a) shows the comparison of implanted diamond substrates pre- and post-annealing. Prior to annealing, only a weak diamond peak from the back substrate and low sp^3 amorphous carbon features are observed at $\approx 1500\,\mathrm{cm}^{-1}$ [13]. Post annealing, three peaks are observed. The $\approx 1595\,\mathrm{cm}^{-1}$ and $\approx 2268\,\mathrm{cm}^{-1}$ peaks are strongly correlated with high sp^3 amorphous carbon composite [13], while the $\approx 1325\,\mathrm{cm}^{-1}$ peak represents the partially recovered top membrane layer [14].

Figure S2 (b) shows the Raman peak of a $110\,\mathrm{nm}$ thick, $\approx 250\,\mathrm{nm}$ overgrowth diamond membrane grown in high temperature condition for $40\,\mathrm{h}$. The sample is named as "N doped membrane" because of the in-situ doping of ^{15}N during overgrowth. The Raman linewidth of this membrane ($1.486(14)\,\mathrm{cm}^{-1}$) is slightly broader than the low temperature one. It is likely that this slight broadening of $0.11\,\mathrm{cm}^{-1}$ originates from the higher heterogeneity of the crystal quality due to slight change in growth conditions from the delta doping and higher synthesis temperatures.

2.3 Membrane thickness and growth rate characterization

In this work, we applied two different approaches to ascertain the thicknesses of the membranes. For the growth rate analysis and thickness-insensitive applications, we used a profilometer (Bruker DektakXT) to measure the height difference between the diamond membranes and the carrier wafers supporting them. For thickness-sensitive applications, we use an ellipsometer (Horiba UVISEL2) to optically monitor the thickness during etching processes.

For the profilometry characterizations the height limit is set to $6.5\,\mu m$ and the typical scanning length is set to $500\,\mu m$ with scanning time $\approx 200\,\mathrm{s}$. The scanning trajectory covers the full length of the membrane for a more accurate value. This measurement is launched after the HSQ annealing and before the "Ar/Cl_2" etching. According to the characterization results, transferred membranes without overgrowth are $340(5)\,\mathrm{nm}$ thick. The thicknesses of the $40\,\mathrm{h}$ overgrown membranes are $680\,\mathrm{nm}$ to $740\,\mathrm{nm}$ (low growth temperature of $500\,°\mathrm{C}$) and $570\,\mathrm{nm}$ to $600\,\mathrm{nm}$ ($700\,°\mathrm{C}$ growth temperature), resulting in growth rates of $9.3(8)\,\mathrm{nm\,h}^{-1}$ and $6.2(4)\,\mathrm{nm\,h}^{-1}$, respectively (growth rate uncertainty from multiple membranes measured). For thickness-insensitive applications, the ICP etching recipe for each membrane is calculated from measured initial thickness, the target thickness, and the ICP etching rates (Table S2).

We also used profilometry characterization to ascertain the overall membrane height and flatness. A scan across a ^{15}N-doped diamond membrane after the transfer is shown in Figure S5 (e). The result indicates an average membrane thickness of $603(10)\,\mathrm{nm}$ before the back etching. This value shouldn't be affected by the back etching process as the ICP etching rate is uniform within the area of the membrane. Due to the angle between the membrane edge and the scanning direction, the length of the membrane in this figure seems to be longer than $200\,\mu m$. Since the scanning result includes some local height variation on the membrane, the real flatness should be less than $10\,\mathrm{nm}$, which indicates a very uniform thickness across the whole membrane area.

This excellent uniformity is crucial for future device fabrication and potential applications.

For thickness-sensitive applications, we used the ellipsometry scanning to the diamond membranes. This technique was firstly applied in nanophotonic integration studies[15]. The scanning area is set to $85\,\mu\mathrm{m} \times 35\,\mu\mathrm{m}$ and is carefully aligned to the membrane surface. The scanning step is set to $\approx 0.05\,\mathrm{eV}$ with long probing time at each step ($\approx 1\,\mathrm{s}$) to reduce the fitting error. To fit the diamond layer, we use Cauchy's equation for transparent material

$$n(\lambda) = A + \frac{B \cdot 10^4}{\lambda^2} + \frac{C \cdot 10^9}{\lambda^4}, \tag{1}$$

where $A = 2.378$, $B = 1.300$ and $C = 0.000$, extracted from[16]. By using this model, we are able to get a $\leq 4\,\mathrm{nm}$ height uncertainty for $\leq 50\,\mathrm{nm}$ membranes.

2.4 HSQ fluorescence analysis and vapor HF treatment

As the adhesion layer between diamond membranes and carrying wafers, the HSQ introduces extra fluorescence under laser excitation. These additional counts mostly span from $560\,\mathrm{nm}$ to $850\,\mathrm{nm}$ and can affect the PL measurements to some extent, depending on the types of color centers being measured.

The resonant PLE measurements are not affected due to their narrow optical linewidths, which allows for the use of exceptionally low excitation power ($\leq 100\,\mathrm{nW}$) and realize a large contrast of ≈ 30 against the background and APD dark counts. Therefore, for experiments and technologies that rely on resonant interactions, such as those proposed for quantum networking, the HSQ background will have minimal impact on device performance. The off-resonant photoluminescence measurements on group IV color centers (such as GeV^-) are mainly used for identifying the center locations, and are not critical for their performance in quantum networking. Due to the high Debye-Waller factors, group IV centers are typically measured with a narrow bandpass filter, which filters out most of the fluorescence from HSQ. With $10\,\mathrm{mW}$ excitation ($532\,\mathrm{nm}$) and appropriate filter sets, the HSQ fluorescence is $\approx 10\,\mathrm{kHz}$ to $20\,\mathrm{kHz}$, which is less than the ZPL counts from individual centers ($\approx 20\,\mathrm{kHz}$ to $40\,\mathrm{kHz}$, shown in Figure 3(b) of the main text). Therefore, the presence of HSQ does not fundamentally hinder the optical characterization of GeV^- centers or preclude the usage of GeV^- in quantum networking applications, the primary technology driver for group IV color centers. Despite its limited impact, the HSQ background can be further reduced as wish via membrane suspension ($\approx 2\,\mathrm{kHz}$ to $5\,\mathrm{kHz}$) or vapor HF undercut ($\approx 0.5\,\mathrm{kHz}$ to $1.5\,\mathrm{kHz}$).

For NV^- centers, the usage of a single longpass filter ($561\,\mathrm{nm}$) leads to a relatively high HSQ counts ($300\,\mathrm{kHz}$ to $500\,\mathrm{kHz}$ under $10\,\mathrm{mW}$ green laser excitation), which hinders the PL and ODMR measurements. Therefore, NV^- measurements benefit from reducing the HSQ background counts. In this work, we combined the suspended membrane fabrication (discussed in section 1.8) and the vapor HF treatment (discussed below) to reduce the total background to $15\,\mathrm{kHz}$ to $30\,\mathrm{kHz}$, which is comparable to the bulk diamond background. Other potential methods to reduce the HSQ background, such as HSQ patterning before transfer, usage of tri-acid clean to oxidize the HSQ layer, or material substitution with less optical fluorescence, will be explored in future studies.

The vapor HF treatment was used optionally to further minimize the background fluorescence from the HSQ underneath the suspended membrane region. During the vapor HF treatment, the chamber pressure is set to $24.5\,\mathrm{Torr}$, with H_2O flow at $18.9\,\mathrm{mL\,min^{-1}}$ and HF flow at $40\,\mathrm{sccm}$. Figure S5 (a) shows a bare carrier wafer after trench fabrication, and Figure S5 (b) shows one of the trenches after the HSQ deposition, annealing, and vapor HF. The image of the wafer prior to vapor HF can be seen in Figure S4 (d), where a colorful interference gradient can be observed. By carefully tuning the process duration ($2\,\mathrm{s}$), we are able to remove most of the HSQ on the sample, while leaving the thermal oxide layer almost untouched. Thus the HSQ has a negligible contribution to the background fluorescence under laser excitation. The sample shown in Figure 3 (a) of the main text received an excess HF treatment ($10\,\mathrm{s}$). The zoomed in image is shown in Figure S5 (c), where an undercut region (rainbow color) with $\approx 18\,\mathrm{\mu m}$ width can be seen, as well as some undesired surface patterns on the SiO_2 - Si carrier wafer (dark dots). Those unwanted effects are largely suppressed by shortening the process duration to $2\,\mathrm{s}$, as seen in Figure S5 (d). The undercut width is $\approx 5\,\mathrm{\mu m}$ with no visible damage on the carrier wafer.

2.5 Dopant and isotope characterization via SIMS

The quality of the isotopic enrichment (replacement of naturally abundant ^{13}C with ^{12}C) is characterized via SIMS (EAG labs) and a typical carbon isotope scan is shown in Figure S9 (a). We reproducibly achieve $99.99\,\mathrm{at.\%}$ ^{12}C diamond during overgrowth, a two orders of magnitude reduction compared to natural abundance as shown in Figure S9 (a). This is currently limited only by the isotopic enrichment of the precursor CH_4 gas.

Nitrogen doping effectiveness as a function of nitrogen precursor gas flow rate was also characterized via SIMS (EAG labs). Characterization of this was done via multiple scans on multiple delta doped samples with specific nitrogen doped layers (multiplicity for statistics). Fitting of the Gaussians (given the delta-doping layer thickness, a Gaussian serves as a valid approximation for the nitrogen distribution) was used to quantify the nitrogen density, which was only restricted by the depth distribution of these delta-doped layers. The FWHM is then used as an approximation of the delta-doping layer thickness, whereas the amplitude is used as an approximation of the atomic density ($\mathrm{cm^{-3}}$) within that layer. A typical characterization run for a ^{15}N triple delta-doped diamond is presented in Figure S9 (b). It remains to be noted that we also regularly characterize the presence of ^{14}N to look for contamination, however, this value is regularly below the SIMS detection limit of about $2 \times 10^{-15}\,\mathrm{cm^{-3}}$.

3 group IV center characterization

3.1 Experimental setup for group IV center measurements

The membrane samples are mounted in a closed-loop cryostation (Montana S200) for low temperature measurements. The sample temperature during the measurements is $5.38\,\mathrm{K}$ as ascertained by a sensor (Lakeshore Cryotronics) installed directly on the sample mount. The position

of the membrane is controlled by three closed-loop piezo positioners (Attocube ANC 350). The incident light beams are navigated by a fast steering mirror (Newport FSM-300) controlled by an analog output device (National Instruments, PCIe-6738). For the PL measurements, we use a $532\,\mathrm{nm}$ continuous wave (CW) laser (Lighthouse Photonics Sprout-G) as the excitation source. For photoluminescence excitation (PLE) measurements of GeV^-, the excitation laser is generated by a wave mixing module (AdvR Inc.) combining a tunable CW Ti:Sapphire laser (M Squared Solstis) and a monochromatic CW laser in telecommunication band (Thorlabs, SFL 1550P). We use this laser to scan across the GeV^- ZPL wavelength (C line) and collect phonon sideband counts. At the collection ports, we use a $50 : 50$ cube beam splitter (Thorlabs Inc. BS013) to divide signals to two branches for counts collection and spectroscopy measurements. A single photon counting module (SPCM) (Excelitas Technologies) is mounted to the counting beam, while a $1200\,\mathrm{gr\,mm^{-1}}$ spectrometer (Princeton Instruments, SpectraPro HRS) is fiber coupled to the spectra beam. The camera of the spectrometer (Princeton Instruments, PIXIS) is maintained at $\leq -70\,°\mathrm{C}$ to minimize background counts. The spectrometer branch can also be connected to another SPCM to perform autocorrelation measurements. We use a combination of bandpass filters (Semrock FF01-615/24-25, Semrock FF01-600/14-25) for GeV^- PL measurements. When performing PLE measurements, we collected the phonon sidebands via double filters ($2\times$ Semrock FF01-647/57-25). All lenses in the confocal setup are achromatic doublets with AB coatings (Thorlabs Inc.) to reach maximum transmission efficiency over broadbands. The PL map obtains a very low background fluorescence ($\approx 1500\,\mathrm{Hz}$) even under $10\,\mathrm{mW}$ excitation, as shown in Figure 3 (b) of the main text. The optical setups for SiV^- and SnV^- characterizations are almost identical, except for the bandpass filter (Semrock FF01-740/13-25 for SiV^- and a Semrock FF01-615/24-25 for SnV^-). Since GeV^- centers are also present in the PL map of SnV^- centers, we use the spectrometer to identify the center type before proceeding with further characterizations.

3.2 GeV^- creation efficiency

We estimate the creation yield of GeV^- by counting the number of fluorescent centers in a certain area. Given the good signal-to-background ratio of ≈ 30, any resolvable centers when counting each $20\,\mathrm{\mu m}$ by $20\,\mathrm{\mu m}$ PL map were included in our counting. The average number of centers per areal spot is $53(3)$ ($\approx 0.13\,\mathrm{\mu m^{-2}}$), yielding a creation yield of $6.6(4)\,\%$ from the implantation dose ($2\times 10^8\,\mathrm{cm^{-2}} \approx 2\,\mathrm{\mu m^{-2}}$). This yield is higher than the previous reported value[17]. We note the high signal-to-background ratio can improve the visibility of darker GeV^- centers, which potentially increase the estimated yield.

3.3 Spectra of Group IV centers and GeV auto-correlation measurements

The GeV^- membrane sample were co-doped with Si^+ and Sn^+ with the same density and target depth. Figure S6 (a-c) shows the representative spectra of GeV^-, SiV^- and SnV^- centers under $5.4\,\mathrm{K}$. Like GeV^- centers, the ZPL peaks of SiV^- and SnV^- are comparable with the bulk diamond values[18,19]. The A and B peaks of SnV^- are not resolvable due to large spin-orbit

coupling and limited phonon population under such a low temperature. More in-depth studies of SiV^- and SnV^- centers are left for subsequent work to explore.

Off- and on-resonance measurements are performed on GeV^- centers, as shown in Figure S6 (d). Both curves exhibit anti-bunching features with $g^{(2)}(0)$ well below 0.5, originated from their single center nature. During PL auto-correlation measurements, the off-resonance laser ($532\,\mathrm{nm}$) is operated at a slightly lower power ($3\,\mathrm{mW}$) to avoid bunching effects. The PLE auto-correlation measurement indicates a clear Rabi oscillation pattern induced by an on-resonance excitation.

The reasons for the non-zero $g^{(2)}(0)$ values ($0.17(2)$ for PL and $0.19(5)$ for PLE) depend on the specific type of measurements. For PL autorrelation measurements, we observed a slowly decreasing ZPL signal with respect to the measurement time. This can be explained by a gradually changing surface termination with the presence of the excitation laser, which affects the preferred electronic configuration of GeV^- centers. As a result, the color centers may experience more times in their optically dark state, slowly quenching the overall signal. In the future, a more rigorous surface termination scheme, such as a tri-acid clean or oxygen termination can be applied to improve the GeV^- stability. For the PLE autocorrelation measurements, the average ZPL linewidth is ≈ 2.4 times wider than the single scan values. With the frequency of the excitation source fixed, the spectral diffusion causes considerable fluctuations of phonon sideband counts, raising the PLE $g^{(2)}(0)$ value. Future optimizations on the linewidth of the GeV^- emissions can effectively enhance the average signal and suppress the $g^{(2)}(0)$.

3.4 GeV^- emission linewidth and spectral jump analysis

The optical linewidth of GeV^- centers approaches the lifetime limit by a factor of 3 (6) during single (average) scans. While the synthesis of optimized narrow linewidth color centers is beyond the scope of this work, multiple strategies can be introduced to overcome the extrinsic broadening factors. To begin with, the optical coherence is impacted by thermal effects via electron-phonon interactions, as modeled and characterized in[20]. Therefore, narrower linewidths may be achievable at lower characterization temperatures. Additionally, some inhomogeneous broadening is attributed to the implantation-induced damage[21]. In theory, this can be minimized by doping Ge in-situ during the overgrowth process[22], which is left for subsequent studies to explore. Furthermore, optimized surface terminations should contribute to reducing surface charge related noise. We excluded the optical power broadening in this work by characterizing the saturation power and tuning the excitation to be slightly lower than this saturation point before every linewidth measurement.

We also observed the switching phenomena for GeV^- in most membrane samples during PLE measurements. A long term scan ($25\,\mathrm{min}$) of a single GeV^- is shown in Figure S6 (e). This center is located in a $20\,\mathrm{h}$ membrane sample with only Si^+ and Ge^+ implantation. The origin of the switching is currently under investigation.

3.5 GeV$^-$ zero phonon lines distribution

To evaluate the inhomogeneous broadening of the implanted GeV$^-$ centers, we included an electronic grade diamond as a reference sample during the Ge$^+$ implantation and subsequent annealing. Therefore, the reference diamond received identical ion implantation and annealing steps. Under the same cryostation temperature ($5.4\,\mathrm{K}$), we measured a total of 75 GeV$^-$ centers in diamond membranes and 30 centers in the reference sample, shown in Figure S6 (f) left and right. If all centers are included in deriving the inhomogeneous broadening, we find a standard deviation of $\sigma = 1.16\,\mathrm{nm}$ for the membrane and $\sigma = 1.52\,\mathrm{nm}$ for the reference sample. However, these numbers are mostly influenced by $\approx 10\,\%$ of the centers with large wavelength shifts (such as $607\,\mathrm{nm}$). These shifts are possibly induced by local strain distortion from the implantation process, and can be found in both membrane and bulk cases. Moreover,these σ values are expected to have a large uncertainty due to limited sampling. Following a similar analysis [23], confining the ZPL wavelengths of GeV$^-$ centers to within a range between $601.5\,\mathrm{nm}$ and $603.2\,\mathrm{nm}$ (which includes $\approx 90\,\%$ of the centers) may give a better value for comparison. Compared to the inhomogeneous broadening in the reference ($\sigma = 0.18\,\mathrm{nm}$), the values we obtained are $\sigma = 0.18\,\mathrm{nm}$ for the reference diamond and $\sigma = 0.19\,\mathrm{nm}$ for the diamond membranes. These similar inhomogeneous broadening values indicate comparable crystal environments between the diamond membrane and bulk diamond.

4 NV$^-$ characterization

4.1 Experimental setup for NV$^-$ characterization

The room temperature NV$^-$ coherence measurements were performed in a custom-built confocal microscope with a $532\,\mathrm{nm}$, free-space laser (Oxxius 532S-150-COL-PP) and a 100X, 0.9NA air objective (Olympus MPLFLN100X). The laser spot position is controlled with a fast steering mirror (Newport FSM-300) and a 4f lens pair. The laser is gated with an acousto-optic modulator (AOM) (Gooch & Housego) and PL is measured with a PDM photon counter (MPD PD-050-CTD-FC). Microwave pulses are generated by a SRS SG-396, amplified by a Mini-Circuits ZHL-15W-422-S+, and gated by both a Mini-Circuits ZASWA-2-50DR+ RF switch and external amplitude modulation of the signal generator. Pulses are applied to the NV$^-$ centers via a $25\,\mu\mathrm{m}$ Al wire draped over the sample. The experiment timing is handled by a Swabian Pulse Streamer.

A static magnetic field of $15\,\mathrm{gauss}$ is applied at approximately 35° to the [100] sample surface. Given the orientation of the sample, the field is at a 10° angle to the [111] crystal axis.

4.2 NV$^-$ coherence measurements and T$_2$ comparison with delta-doped results from previous studies

We use standard free induction decay (FID) and spin echo (SE) sequences to measure T_2^* and T_2, respectively. Each sequence, shown in Figure S8, is composed of two branches, which measure both the $|0\rangle$ and $|-1\rangle$ projections. After applying a $\pi/2$-pulse on the NV$^-$, we allow

the spin to evolve for a time τ (and apply a π_y pulse at $\tau/2$ for the SE) before applying either another $+\pi/2$-pulse or a $-\pi/2$-pulse to project into the readout basis. The readout windows at the beginning of each laser pulse are binned as the readout signals, $S_{0,-1}$, and the readout window at the end is binned as a reference, R, which allows us to correct for slow drift over each measurement cycle[24]. All measurements are processed as

$$\text{Coherence} = \frac{\mathrm{S}_0 - \mathrm{S}_{-1}}{\mathrm{R}} \cdot \frac{\mathrm{R}[0]}{\mathrm{S}_0[0] + \mathrm{S}_{-1}[0]}, \tag{2}$$

where the second term accounts for absolute count discrepancies between readout at the beginning and end of the laser pulse that arise from the finite rise time of the AOM. Each pulse sequence is a different length due to sweeping τ, but run for the same amount of time overall, necessitating normalization by the reference to cancel this variation. Ultimately this means that shorter-τ data are average over more instances than longer-τ data. We fit T_2^* measurements to the following[25]

$$B + \exp\left[-(t/T2^*)^2\right] \cdot \sum_{i=1}^{2I+1} a_i \cos\left(2\pi f_i(t + \phi_i)\right), \tag{3}$$

Where B is a background offset, a_i, f_i, and ϕ_i are the amplitude, frequency, and phase of each sinusoid corresponding to each hyperfine peak, and I is the nitrogen nuclear spin ($1/2$ for ^{15}N, 1 for ^{14}N) . T_2 measurements are fit to

$$B + A\exp\left[-(t/T_2)^n\right], \tag{4}$$

Where B is an offset, A is the amplitude, and n is either a free parameter or fixed at 3[25], when the former does not fit.

Previous delta-doping work demonstrated T_2 times for deep NV⁻ centers ($\geq$52 nm) on the order of $\approx$700 µs[3], with measured values dropping drastically ($\leq$100 µs) as the delta-doped layer was brought close to the diamond surface ($\leq$35 nm). Indeed, it is expected that surface-related decoherence is a main limitation for the T_2 times presented in this work[12]. The impact of surfaces is further amplified in membranes, as the color centers are in close proximity to two surfaces rather than one.

4.3 NV$^-$ strain measurements

The NV$^-$ center is sensitive to both strain and electric field ($\mathbf{\Pi}$) through spin state coupling, as shown in the Hamiltonian

$$H = (D + \Pi_z)S_z^2 + \Pi_x\left(S_y^2 - S_x^2\right) + \Pi_y(S_xS_y + S_yS_x). \tag{5}$$

Parallel strain/electric field (Π_z) shifts the effective zero-field splitting, while the perpendicular components ($\Pi_{x,y}$) split the upper and lower spin transitions[26]. However, there is a discrepancy in the strength of the couplings for each physical origin. The electric field susceptibilities $d_{\epsilon:\|,\perp}$ =0.35 Hz cm V^{-1} and 17 Hz cm V^{-1}[27], while the strain susceptibilities $d_{\sigma:\|,\perp}$ =13.3 GHz and 21.5 GHz[28]. We thus expect that if local electric fields dominate, the splitting between the

upper and lower transitions should be much larger than the shifts in zero-field splitting, whereas if strain dominates these effects should be of similar magnitudes.

Through zero-field ODMR measurements, we found in all but one case that the splitting was greater than or the same magnitude as the shifts. In Figure S7 (a) we present the shifts in zero-field splitting versus the NV orientation, as determined at non-zero field, to extract an upper bound on the strain in the membrane. If we assume the larger shifts of $\approx 1\,\mathrm{MHz}$ arise entirely from strain – an over-estimate, given the above discussion – we arrive at a strain of $< 10^{-4}$.

Multiple factors can contribute to the built-in strain. For instance, the dry transfer step might introduce in-plane strain due to approach angle and speed. This can be reduced by reducing the approaching speed of the PDMS stamp with a smaller angle. Additionally, although the HSQ layer collapses above $465\,^{\circ}\mathrm{C}$ and potentially releases the strain during the annealing, some residual strain might still remain. Furthermore, due to different thermal expansion ratios between the diamond membrane and carrier wafers, a thermal-induced strain appears when the sample is cooled down to room temperature or at $5.4\,\mathrm{K}$ of PL characterizations. The direction and the magnitude of the strain depend on the material of the carrier wafers. Finally, some on-chip structures (such as trenches) can alter the strain distribution across the membrane.

4.4 NV$^-$ T_1 comparison

We compare T_1 population decay times for two NV$^-$ centers with an order of magnitude separation in coherence times to confirm that a reduction in T_1 is not responsible for the reduction in T_2. Figure S7 (b) shows the differential T_1 measurement for NVX ($T_2 = 13(1)$ µs) and NVY ($T_2 = 146(53)$ µs). Over $5\,\mathrm{ms}$ there is no observable increased decay for NVX, indicating T_1 plays no role in limiting coherence.

4.5 [N] and [NV$^-$] estimation

Both the overgrowth background [^{14}N] and in-situ doped [^{15}N] were determined via calibrated quantitative secondary ion mass spectroscopy (SIMS) characterization (EAG Laboratories). All SIMS derived error bars are standard deviation from a duplicate experimental characterization. The SIMS detection limit of [^{14}N] placed an upper bound on the background nitrogen contamination $4.5(2) \times 10^{15}\,\mathrm{cm}^{-3}$ ($\leq 26(1)\,\mathrm{ppb}$). For the $0.06\,\mathrm{sccm}$ $^{15}N_2$ dopant precursor flow rate used for the generation of in-situ doped δ-doped membranes, we obtain a [^{15}N] of $5.1(10) \times 10^{15}\,\mathrm{cm}^{-3}$ ($30.8(57)\,\mathrm{ppb}$) with an EAG quoted ^{15}N detection limit of $1 \times 10^{15}\,\mathrm{cm}^{-3}$ ($5.7\,\mathrm{ppb}$).

The nitrogen implanted membrane sample was subjected to a $48\,\mathrm{keV}$, $2 \times 10^{8}\,\mathrm{cm}^{-2}$ [N] dose. At this energy, the straggle (standard deviation of implantation as approximated with SRIM) is $14\,\mathrm{nm}$. Assuming the bulk of the nitrogen resides in a $\leq 30\,\mathrm{nm}$ thick region, the expected [N] from the implantation to be $6.7 \times 10^{13}\,\mathrm{cm}^{-3}$ ($\approx 0.4\,\mathrm{ppb}$) within the implanted region. Comparing this to the observed [^{14}NV$^-$] of $1.8 \times 10^{12}\,\mathrm{cm}^{-3}$ ($0.01\,\mathrm{ppb}$) obtained from a typical count of a 10 µm by 10 µm region of the $120\,\mathrm{nm}$ thick membrane. This suggests an upper bound on the N-to-NV conversion efficiency at $\leq 2.5\,\%$. Realistically, in-grown background [N]

are also being converted to [NV$^-$], causing an overestimation of this conversion efficiency. The ambiguity of the [N] origin can be avoided by implanting ^{15}N in future iterations of experiments.

Isotopic tagging of dopant species in the ^{15}N δ-doped membrane was used to provide further insight into this. From a PL survey of a $12\,\mu\text{m}$ by $12\,\mu\text{m}$ area of the $110\,\text{nm}$ thick $delta$-doped membrane (post-irradiation and annealing), we noted a relative ratio of $7 : 11$ for $[^{15}\text{NV}^-]$: $[^{14}\text{NV}^-]$. Knowing that the delta doped area is $\approx 2\,\text{nm}$ thick, with $[^{15}\text{N}] = 5.1(10) \times 10^{15}\,\text{cm}^{-3}$, we can work out the approximate density of the [^{14}N] in the rest of the membrane—assuming a uniform conversion efficiency for both the δ-doped [^{15}N] and background in-grown natural abundance [N] (this approximation can break down easily, but remains useful here). Once geometric effective layer thicknesses are considered, i.e., $2\,\text{nm}$ thick portion of [^{15}N] vs a $110\,\text{nm}$ thick $\approx$ $[^{14}\text{N}]$ membrane, we end up with a normalized observed $[^{15}\text{NV}^-] : [^{14}\text{NV}^-]_{normalized}$ of $7 : 0.2$, resulting in a background [^{14}N] of $0.124(5) \times 10^{15}\,\text{cm}^{-3}$ $(0.70(3)\,\text{ppb})$. A common source of magnetic noise for NV$^-$ centers is unconverted nitrogen atoms (P_1 centers). Taking into account nitrogen originating from both implantation and background in-grown sources, we estimate a density $[N]$ of $\leq 1\,\text{ppb}$. At these concentrations, P_1s should not contribute heavily to the decoherence.[29]

References

[1] B. C. Rose, *et al.*, *Science* **361**, 60 (2018).

[2] P. John, M. D. Stoikou, *Physical Chemistry Chemical Physics* **13**, 11503 (2011).

[3] K. Ohno, *et al.*, *Applied Physics Letters* **101** (2012).

[4] S. A. Meynell, *et al.*, *Applied Physics Letters* **117**, 194001 (2020).

[5] N. Yang, ed., *Novel Aspects of Diamond*, vol. 121 of *Topics in Applied Physics* (Springer International Publishing, Cham, 2015).

[6] J. C. Lee, A. P. Magyar, D. O. Bracher, I. Aharonovich, E. L. Hu, *Diamond and Related Materials* **33**, 45 (2013).

[7] M. Ruf, *et al.*, *Nano Letters* **19**, 3987 (2019).

[8] J. F. Ziegler, M. D. Ziegler, J. P. Biersack, *Nuclear Inst. and Methods in Physics Research, B* **268**, 1818 (2010).

[9] K. J. Brown, E. Chartier, E. M. Sweet, D. A. Hopper, L. C. Bassett, *ACS Chemical Health & Safety* (2021).

[10] K. M. Fu, C. Santori, P. E. Barclay, R. G. Beausoleil, *Applied Physics Letters* **96**, 1 (2010).

[11] A. H. Piracha, *et al.*, *Nano Letters* **16**, 3341 (2016).

[12] S. Sangtawesin, *et al.*, *Physical Review X* **9**, 1 (2019).

[13] A. C. Ferrari, *Solid State Communications* **143**, 47 (2007).

[14] A. P. Magyar, *et al.*, *Applied Physics Letters* **99**, 1 (2011).

[15] A. Butcher, *et al.*, *Nano Letters* **20**, 4603 (2020).

[16] M. N. Polyanskiy, Refractive index database, https://refractiveindex.info.

[17] N. H. Wan, *et al.*, *Nature* **583**, 226 (2020).

[18] S. Meesala, *et al.*, *Physical Review B* **97**, 1 (2018).

[19] M. E. Trusheim, *et al.*, *Physical Review Letters* **124**, 1 (2020).

[20] K. D. Jahnke, *et al.*, *New Journal of Physics* **17** (2015).

[21] S. B. Van Dam, *et al.*, *Physical Review B* **99**, 161203 (2019).

[22] A. Sipahigil, *et al.*, *Physical Review Letters* **113**, 1 (2014).

[23] T. Iwasaki, *et al.*, *Scientific Reports* **5**, 1 (2015).

[24] B. A. Myers, A. Ariyaratne, A. C. Jayich, *Physical Review Letters* **118**, 1 (2017).

[25] L. Childress, *et al.*, *Science* **314**, 281 (2006).

[26] T. Mittiga, *et al.*, *Phys. Rev. Lett.* **121**, 246402 (2018).

[27] E. Van Oort, M. Glasbeek, *Chemical Physics Letters* **168**, 529 (1990).

[28] P. Ovartchaiyapong, K. W. Lee, B. A. Myers, A. C. B. Jayich, *Nature Communications* **5**, 4429 (2014).

[29] Z. H. Wang, S. Takahashi, *Physical Review B - Condensed Matter and Materials Physics* **87**, 1 (2013).

Species	Energy [keV]	Dose [cm^{-2}]	Target depth [nm]	Straggle [nm]
$^{4}He^{+}$	150	5×10^{16}	413	39
$^{14}N^{+}$	48	2×10^{8}	60	15
$^{28}Si^{+}$	58	2×10^{8}	40	11
$^{74}Ge^{+}$	98	2×10^{8}	40	10
$^{120}Sn^{+}$	150	2×10^{8}	42	8

Table S1: Implantation parameters and SRIM simulation results for diamond membrane formation and color center generation.

Recipe name	Ar/Cl_2	O_2/Cl_2	O_2	Al_2O_3 etching
ICP power [W]	400	700	700	400
Bias power [W]	250	100	100	50
Pressure [mTorr]	8	10	10	5
Cl_2 flow [sccm]	40	2	0	0
Ar flow [sccm]	25	0	0	10
BCl_3 flow [sccm]	0	0	0	30
O_2 flow [sccm]	0	30	30	0
Etching rate [$\mathrm{nm\,min^{-1}}$]	$\approx$73	$\approx$177	$\approx$175	$\approx$63 (Al_2O_3)

Table S2: Cl-based ICP etching recipe for diamond. The last recipe, Al_2O_3 etching, is used for hard mask (ALD Al_2O_3) removal during the membrane patterning.

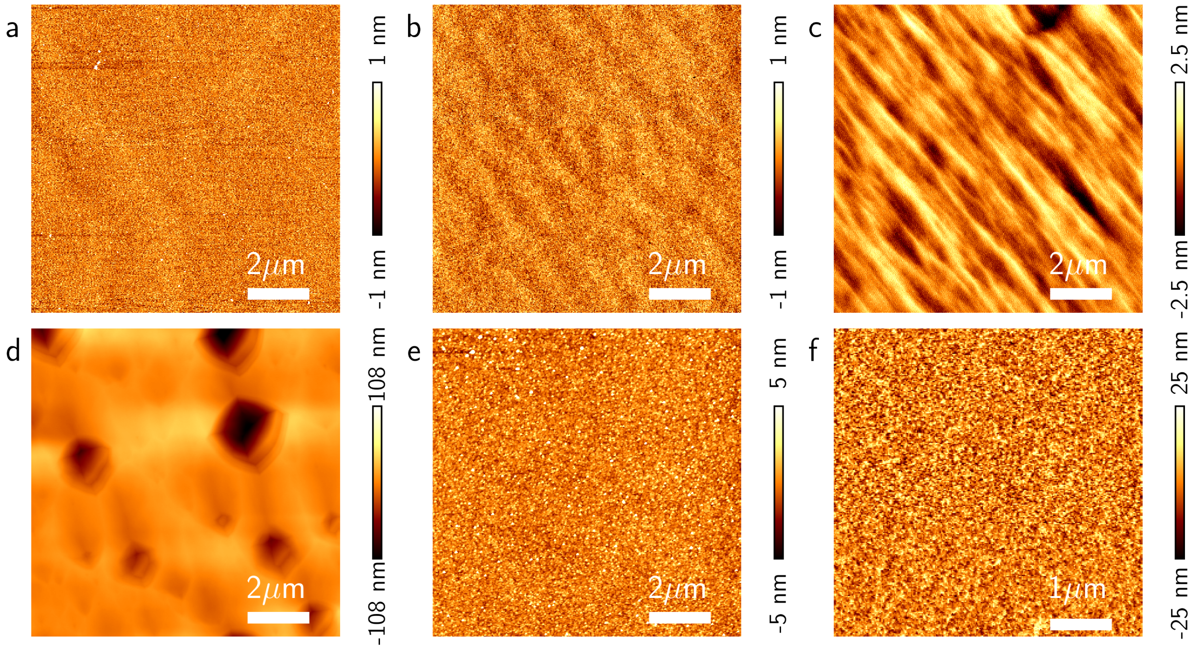

Figure S1: AFM characterizations during diamond membrane synthesis. (a) Fine polished diamond substrate shows $Rq = 0.30\,\mathrm{nm}$. (b) The substrate after implantation and annealing with $Rq = 0.27\,\mathrm{nm}$. (c) The $40\,\mathrm{h}$ overgrown sample with $60\,\mathrm{min}$ hydrogen etch. $Rq = 0.69\,\mathrm{nm}$. (d) The $40\,\mathrm{h}$ overgrown sample with insufficient surface cleaning prior to the growth. $Rq = 18.6\,\mathrm{nm}$. (e) Etched side of the membrane after the dry transfer. $Rq = 1.17\,\mathrm{nm}$. (f) Etched side of the membrane after $30\,\mathrm{s}$ of oxygen ICP with no prior "Ar/Cl_2" etching. $Rq = 6.78\,\mathrm{nm}$.

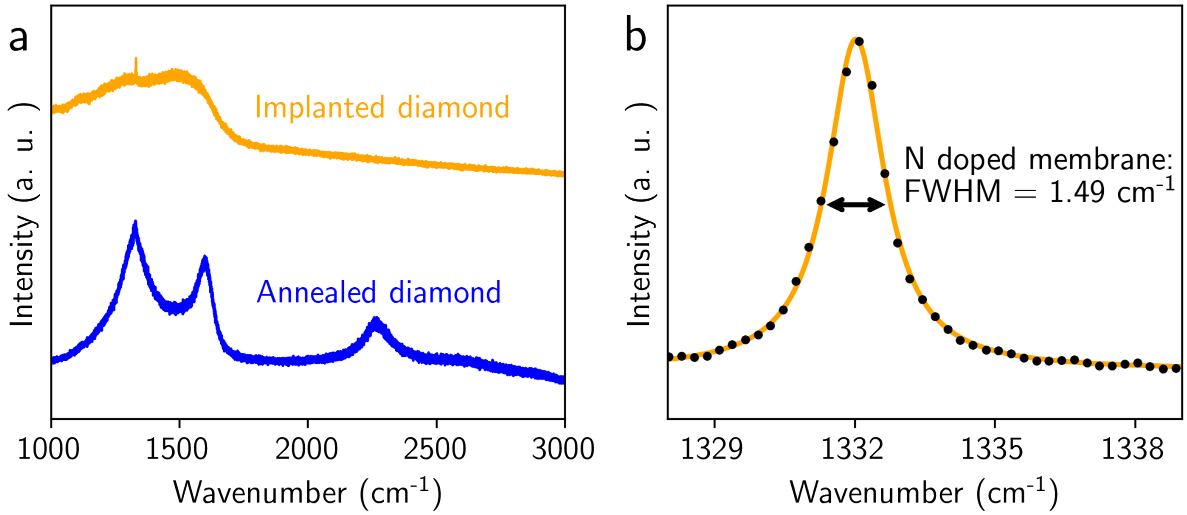

Figure S2: Additional Raman spectroscopy for membrane formation and overgrowth. (a) Orange curve: the implanted diamond substrate prior to annealing. Blue curve: the annealed diamond substrate. (b) The Raman peak for an isotopically purified, $\approx 250\,\mathrm{nm}$ overgrown membrane at $700\,°\mathrm{C}$ ($40\,\mathrm{h}$ growth, back-etched down to $110\,\mathrm{nm}$). The peak is at $1332.03(30)\,\mathrm{cm}^{-1}$, with linewidth $1.486(14)\,\mathrm{cm}^{-1}$.

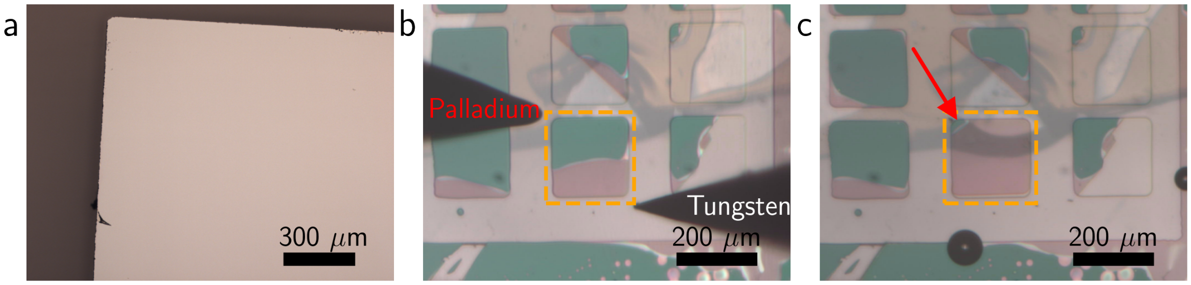

Figure S3: Microscope images for annealed and undercut diamond membranes. (a) The diamond membrane after He^+ implantation and subsequent annealing. The sample exhibits uniform light pink reflections, which can be interpreted as interference effect between a transparent top surface and a reflective graphitic underlayer. (b-c) A half- (almost-) undercut membrane (dashed orange square) at the EC etching step. Both images were taken when the membrane was in DI water. The left (right) electrode in (b) represents the palladium (tungsten) tip. The red arrow in (c) points to the membrane tether. The opaque graphitic layer exhibits interference colors (green), while the undercut region is almost transparent. The plate on the upper right indicates a leftover tether where the membrane has been picked up, while the membranes on the top and right were by-caught during the transfer. Such by-caught phenomena were caused by undesired EC etching due to low resistance of tap water and has been largely suppressed since the DI water is applied instead (See the membrane on the left in both images).

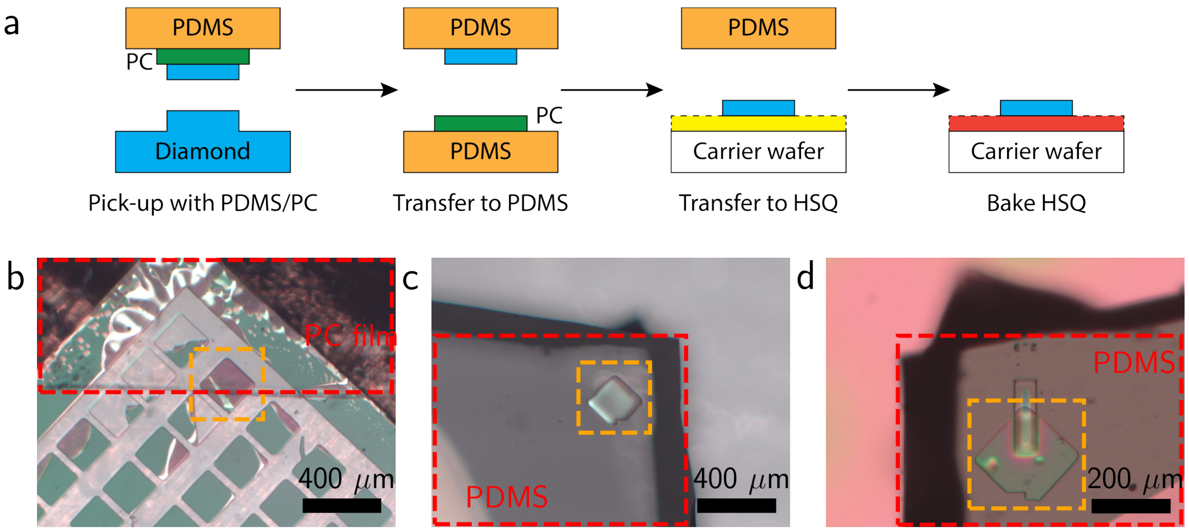

Figure S4: Deterministic diamond membrane transfer. (a) General transfer process flow. The diamond is flipped during the process in order to remove the original membrane afterwards. (b) The PDMS/PC stamp approaches the diamond membrane. The PC film (red dashed rectangle) is located under a PDMS stamp which spans beyond this image. The target membrane (orange dashed square) is linked to the diamond substrate via a small tether (See S3 (c)). (c) The membrane transferred to the PDMS-only stamp (red dashed rectangle) in the second step. (d) The membrane attached to a HSQ-coated trench on a thermal oxide wafer. The bumps under the membrane can be avoided by cleaner PDMS/PC stamp preparation in the future.

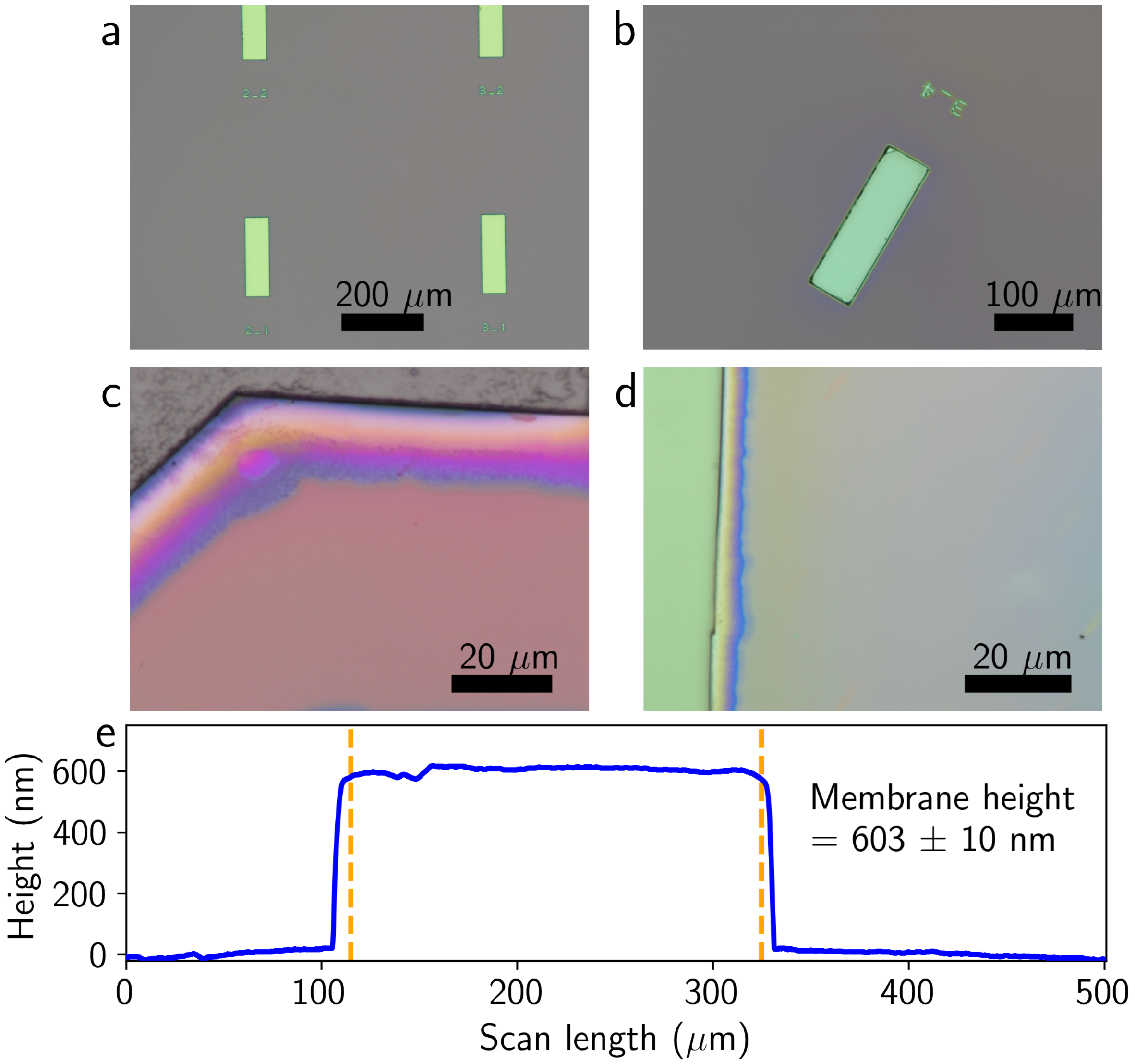

Figure S5: Carrier wafer preparation, vapor HF treatment and membrane thickness characterization. (a) 280 nm SiO_2−Si thermal oxide wafer with 5 μm deep trenches and markers. The silicon (green) exhibits different color from the oxide layer (gray). (b) A trench on the thermal oxide wafer after HSQ spinning, annealing and vapor HF treatment. The HSQ is mostly removed while the thermal oxide layer is barely untouched. (c-d) The undercut edge of the diamond membrane after 10 s (2 s) of vapor HF etching. (e) Profilometry characterization of the membrane thickness after transfer and before back etching. The region of the transferred membrane is indicated by the dashed orange lines. The average thickness for this ^{15}N-doped membrane is 603 nm with a standard deviation of 10 nm.

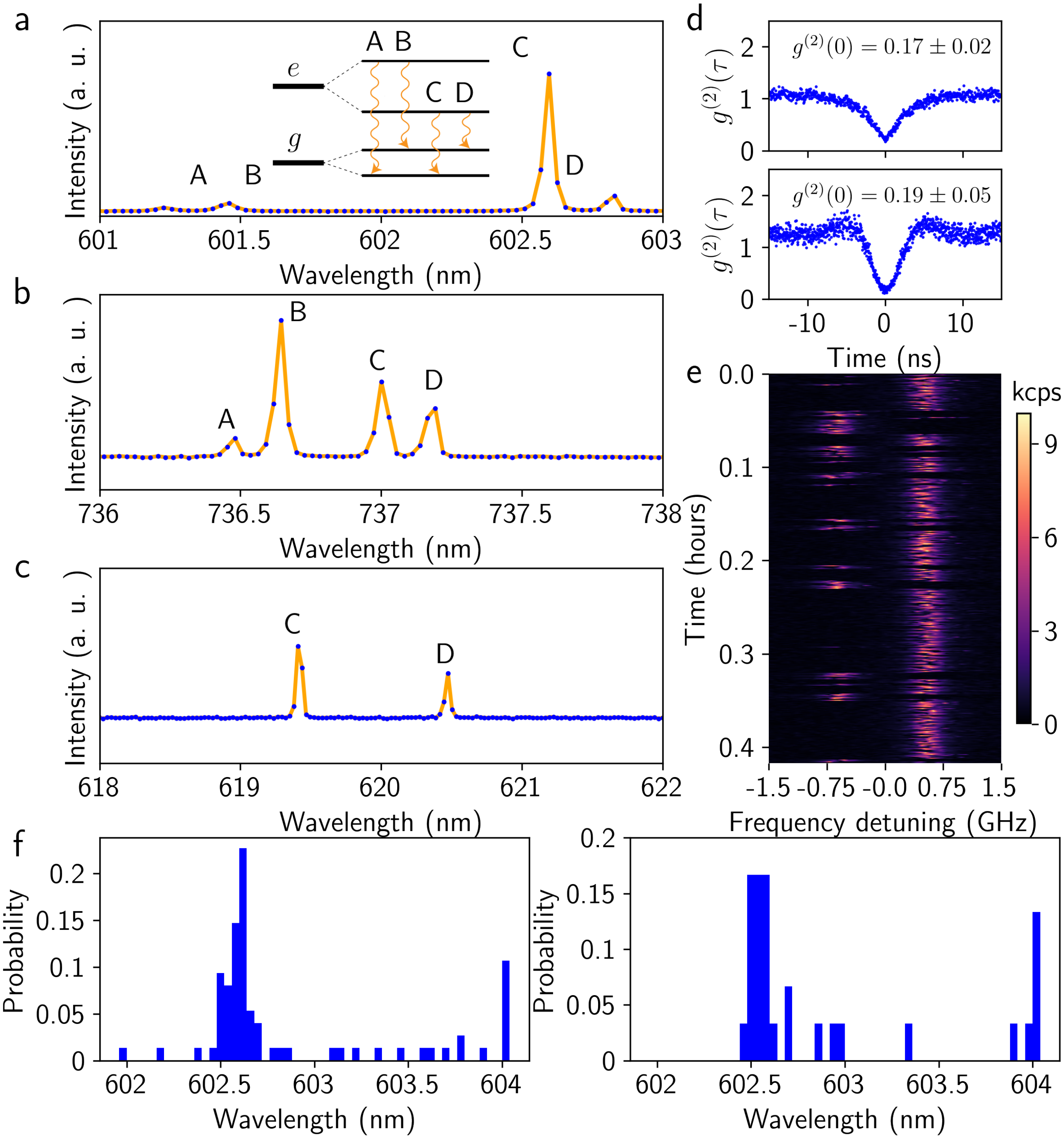

Figure S6: Additional optical measurements of group IV centers. (a-c) The spectra of a representative GeV (SiV^-, SnV^-) center measured under $5.4\,\mathrm{K}$, with $10\,\mathrm{mW}$ $532\,\mathrm{nm}$ laser excitation. The small plot inside (a) represents the general energy levels of negatively charged group IV centers. (d) Off- (on)-resonance autocorrelation measurements of a GeV^- center. The excitation power of the off-resonance laser is $3\,\mathrm{mW}$, while the on-resonance laser is $40\,\mathrm{nW}$. (e) A $25\,\mathrm{min}$ scanning of a spectrally switching GeV^- center. (f) ZPL distributions of GeV^- centers in the diamond membrane (left) and bulk diamond (right).

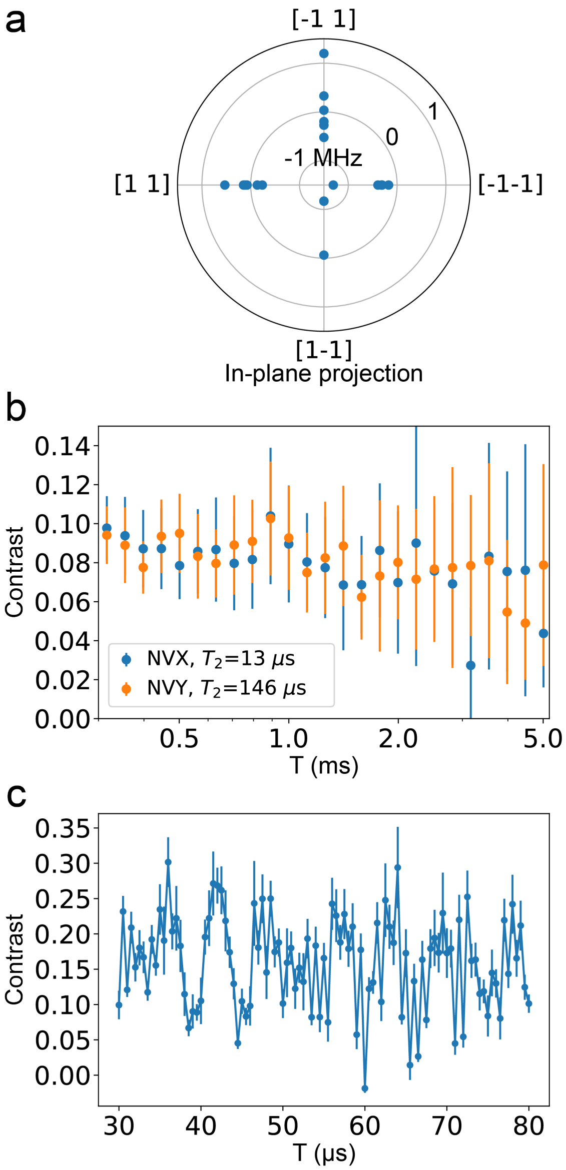

Figure S7: (a) Zero-field splitting of measured NV⁻ centers. (b) T_1 measurement of two NV⁻s with order of magnitude spread in T_2 times. (c) Finer resolution spin echo measurement on the NV⁻ presented in Fig. 4 (a), showing electron spin echo envelope modulation, which is aliased in the main text data.

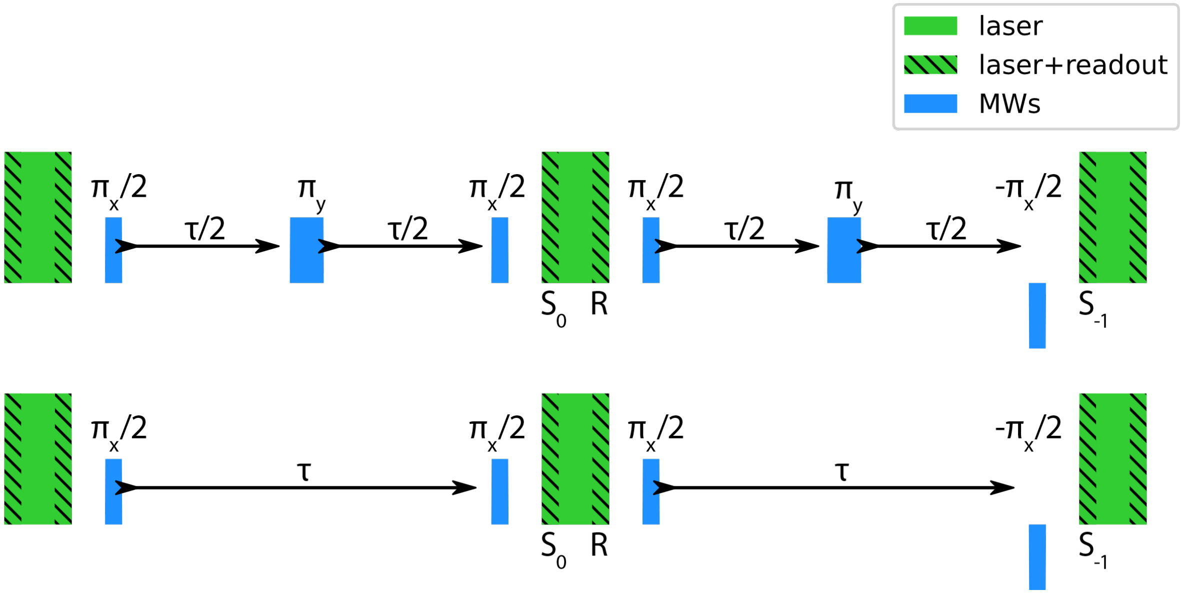

Figure S8: Coherence measurement sequences. Both the spin echo (a) and free induction decay (b) sequences have readout, initialization, state preparation, and state projection pulses. The spin echo sequence has a central π-pulse to refocus the spin.

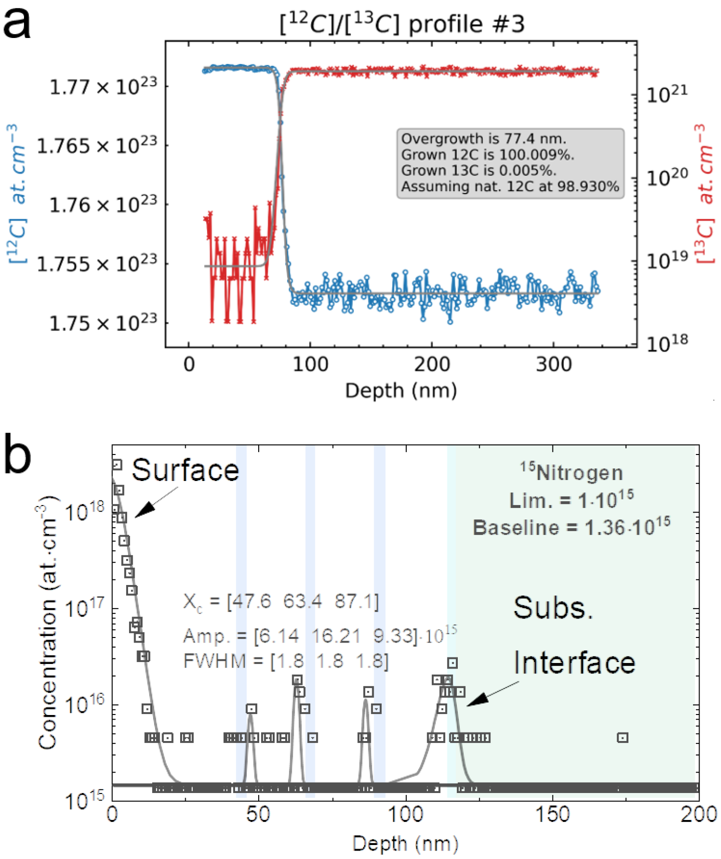

Figure S9: SIMS characterizations. (a) SIMS characterization of a typical isotopically enriched diamond growth using our standard overgrowth showing $99.99\,\mathrm{at.}\%$ ^{12}C diamond. (b) Nitrogen doping effectiveness ^{15}N (background contamination ^{14}N not shown but done parallel to this) for a triple delta-doped overgrowth sample. Fitting of the Gaussians was used to quantify the nitrogen density. The FWHM is used as an approximation of the delta-doping layer thickness, whereas the amplitude is used as an approximation of the atomic density (cm^{-3}) within that layer. The detection limit is shown in graph. We also note a significant amount of nitrogen at the substrate/overgrowth and surface interfaces with ratios consistent of natural abundance of ^{15}N in atmospheric nitrogen (measured via peak area ratio).
